# Motor protein KIF13B orchestrates hepatic metabolism to prevent metabolic dysfunction-associated fatty liver disease

**DOI:** 10.1186/s40779-025-00594-3

**Published:** 2025-03-04

**Authors:** Guo-Lin Miao, Wen-Xi Zhang, Yi-Tong Xu, Yi-Ran Liu, Ping-Ping Lai, Jia-Bao Guo, Gong-Lie Chen, Jing-Xuan Chen, Zi-Hao Zhou, Yan-Wei Li, Chong Zhang, Yang Ding, Lian-Xin Zhang, Yu-Fei Han, Jin-Xuan Chen, Jing-Dong Wu, Yin-Qi Zhao, Si Mei, Yang Zhao, Yuan-Wu Ma, Ling Zhang, Wei Huang, Dong-Yu Zhao, Er-Dan Dong, Yu-Hui Wang, Xun-De Xian

**Affiliations:** 1 Institute of Cardiovascular Sciences, State Key Laboratory of Vascular Homeostasis and Remodeling, School of Basic Medical Sciences, Peking University, Beijing, 100191 China; 2 Department of Cardiology and Institute of Vascular Medicine, Peking University Third Hospital, Beijing, 100191 China; 3 Department of Biomedical Informatics, State Key Laboratory of Vascular Homeostasis and Remodeling, School of Basic Medical Sciences, Peking University, Beijing, 100191 China; 4 Department of Infectious Diseases, Shengjing Hospital of China Medical University, Shenyang, 110134 China; 5 State Key Laboratory of Natural and Biomimetic Drugs, Ministry of Education, Key Laboratory of Cell Proliferation and Differentiation, Beijing Key Laboratory of Cardiometabolic Molecular Medicine, Center for Life Sciences, Institute of Molecular Medicine, College of Future Technology, Peking University, Beijing, 100871 China; 6 Key Laboratory of Human Disease Comparative Medicine, National Health Commission of China (NHC), Beijing Engineering Research Center for Experimental Animal Models of Human Critical Diseases, Institute of Laboratory Animal Science, Chinese Academy of Medical Sciences, Peking Union Medicine College, Beijing, 100021 China; 7 Research Center for Cardiopulmonary Rehabilitation, University of Health and Rehabilitation Sciences Qingdao Hospital (Qingdao Municipal Hospital), School of Health and Life Sciences, University of Health and Rehabilitation Sciences, Qingdao, 266113 Shandong China; 8 Beijing Key Laboratory of Cardiovascular Receptors Research, Peking University Third Hospital, Beijing, 100191 China

**Keywords:** Kinesin family member 13B, AMP-activated catalytic subunit alpha 1, Mitochondrial homeostasis, Lipid metabolism, Metabolic dysfunction-associated fatty liver disease

## Abstract

**Background:**

Kinesin family member 13B (KIF13B), a crucial motor protein, exerts multiple cellular biological functions. However, the implication of KIF13B in metabolic dysfunction-associated fatty liver disease (MAFLD) has not been explored yet. This study aimed to investigate KIF13B’s role and underlying mechanism in MAFLD and proposes it as a potential pharmacological target.

**Methods:**

We assessed KIF13B expression in MAFLD patients and rodent models. The roles of *Kif13b* in lipid metabolism and MAFLD were investigated using whole-body *Kif13b* knockout mice, hepatocyte-specific *Kif13b*-deficient mice and hamsters exposed to different diets. The underlying mechanisms by which *Kif13b* governed hepatic lipid homeostasis and MAFLD progression were explored in vitro. Finally, the *Kif13b*’s impact on atherosclerotic development was studied in the context of MAFLD.

**Results:**

KIF13B expression was reduced in patients and murine models with MAFLD. Rodents with global or liver-specific knockout of the *Kif13b* gene exhibit spontaneous hepatic steatosis, which is further exacerbated by different overnutrition diets. Overexpression of human KIF13B by lentivirus effectively prevented metabolic dysfunction-associated steatohepatitis (MASH) in methionine-choline-deficient diet (MCD)-fed mice. Furthermore, *Kif13b* deficiency accelerates atherosclerosis in the context of MAFLD. Mechanistically, *Kif13b* depletion increases hepatic lipid synthesis and impairs mitochondrial oxidative phosphorylation. Further screening reveals that Kif13b interacts with AMP-activated catalytic subunit alpha 1 (AMPKα1) to regulate the phosphorylation of AMPKα1, governing mitochondrial homeostasis and suppressing sterol regulatory element binding protein 1 (Srebp1)-mediated de novo lipogenesis in the liver.

**Conclusion:**

This work establishes a causal relationship between KIF13B deficiency and MAFLD, emphasizing KIF13B as a potential therapeutic target for treating MAFLD.

**Supplementary Information:**

The online version contains supplementary material available at 10.1186/s40779-025-00594-3.

## Background

Metabolic dysfunction-associated fatty liver disease (MAFLD) is a severe global health concern, accounting for 4% of fatalities over the world annually [1, 2]. MAFLD progresses from metabolic dysfunction-associated fatty liver (MAFL) to the more pathologically severe metabolic dysfunction-associated steatohepatitis (MASH), which can lead to cirrhosis, hepatocellular carcinoma, and a series of systemic metabolic disorders. Notably, MASH can regress to isolated steatosis or persist in a relatively constant state progressing towards cirrhosis, emphasizing the need to control MAFLD progression from its steatosis stage [3]. Even with the recent approval of the thyroid hormone receptor agonist resmetirom by the Food and Drug Administration (FDA) for the treatment of noncirrhotic MASH with moderate to advanced liver fibrosis (consistent with stages F2 to F3 fibrosis), the urgent need to develop new drugs to address the global prevalence of MAFLD has become increasingly evident. The heterogeneous and complex etiologies and pathologies associated with MAFLD present a significant challenge.

Hepatic steatosis arises from an imbalance in liver lipid handling, where fatty acid uptake and de novo lipogenesis (DNL) surpass oxidation and export [4]. Mitochondrial metabolism plays a crucial role in liver lipid breakdown. Improving mitochondrial respiratory activity in the liver can enhance fatty acid degradation, preventing their accumulation and disease progression. Progressive MAFLD is linked to complex lipid metabolic issues and mitochondrial defects, such as mitochondrial biogenesis and loss of cristae in mitochondria [5, 6]. Several human studies have demonstrated a decline in hepatic mitochondrial functionality in MAFLD [7, 8]. However, the therapeutic potential of enhancing mitochondrial metabolism in MAFLD is underexplored due to concerns about increased reactive oxygen species (ROS) from increased β-oxidation. Therefore, it is necessary to identify a secure and efficient target that can improve mitochondrial function without triggering oxidative stress.

Kinesins represent a superfamily of mechanochemical enzymes that bind to microtubules, hydrolyze adenosine triphosphate (ATP), and either transport cargoes along the microtubule tracks or participate in controlling microtubule dynamics inside a cell [9–11]. Kinesin-3 is one of the most prominent families among the kinesin superfamily, consisting of 5 subfamilies (KIF1, KIF13, KIF14, KIF16, and KIF28) [12], and plays critical roles in intracellular transport, endocytosis, signaling, and cell division [13]. Defects in kinesin-3 can cause a spectrum of disorders, such as developmental defects, neurodegenerative disorders, and cancer [14–16]. As a vital member of the kinesin-3 family, KIF13B possesses a highly conserved about 350 amino acid catalytic motor domain responsible for microtubule binding, ATP hydrolysis, and mitochondrial transport, and mediates cytoskeletal crosstalk by interacting with the components of actin and microtubule to regulate mitochondrial functions [17–19]. Recently, it has been reported that KIF13B participates in low density lipoprotein (LDL) receptor related protein 1 (LRP1) endocytosis on the sinusoidal plasma membrane of hepatocytes [20]. Furthermore, Kif13b^−/−^ mice exhibited increased serum cholesterol levels compared with wild-type (WT) mice [20, 21]. Given that the role of LRP1 in lipid metabolism and MAFLD has been well documented [22, 23], we postulated that Kif13b may play a vital role in MAFLD. However, the role of Kif13b in hepatic lipid metabolism and potential development of MAFLD has yet to be documented. This study aimed to examine the function and precise molecular pathways of Kif13b in MAFLD, with the goal of identifying effective therapeutic targets and medications for the treatment of MAFLD in clinical practice.

## Methods

Detailed methods are provided in the Additional file 1: Methods for all procedures carried out in this study. Biological replicates are incorporated for all datasets in this study.

### Human sample collection

This study received approval from the Medical Ethics Committee of Shengjing Hospital at China Medical University (2023PS913K). All participants provided written informed consent.

Human liver samples were collected from 5 healthy controls and 19 patients with MAFLD who underwent percutaneous liver biopsy in the Department of Infectious Diseases at Shengjing Hospital. Histological specimens were scored according to the composite scoring system [steatosis, activity, and fibrosis (SAF) score] [24]. Briefly, the SAF score was calculated as the sum of 3 histological components, including steatosis (grades 0–3), activity (grades 0–4) [hepatocyte ballooning (grades 0–2), lobular inflammation (grades 0–2)], and fibrosis (grades 0–4).

The diagnostic criteria for MAFLD are based on evidence of hepatic steatosis (detected by liver biopsy, imaging or through the use of blood biomarkers and scores), and the coexistence of overweight or obesity, type 2 diabetes mellitus or metabolic dysregulation. The diagnosis criteria of MASH are based on the following criteria: (1) intake of less than 20 g/d alcohol; (2) biopsy proven steatohepatitis; steatosis, inflammatory infiltrates, and ballooning degeneration with or without Mallory bodies or pericellular/perivenular fibrosis; (3) appropriate exclusion of other liver diseases.

The inclusion criteria for healthy controls were as follows: volunteers were required to be free of metabolic abnormalities and liver injury. The inclusion criteria for patients were as follows: the diagnosis must meet the criteria for a MAFLD or MASH diagnosis.

The exclusion criteria for this study encompassed hepatitis C virus infection, autoimmune hepatitis, hepatomegaly, drug-induced liver injury, and total parenteral nutrition, inflammatory bowel disease, celiac disease, hypothyroidism, Cushing’s syndrome, β-lipoprotein deficiency, lipoatrophic diabetes mellitus, Mauriac’s syndrome, and other specific conditions that could lead to fatty liver.

### Animals

Eight-week-old WT (C57BL/6J) male mice and Golden Syrian hamsters were purchased from Vital River Laboratories (Beijing, China). Low-density lipoprotein receptor-deficient (LDLR^−/−^) and leptin-deficient (ob/ob) mice were purchased from GemPharmatech (Nanjing, China). All animals were maintained in a specific pathogen-free standard facility with ad libitum access to a standard chow diet (CD) and water unless otherwise described. All experiments were allowed and followed the guidelines of the Institutional Animal Care and Use Committee of Peking University and were carried out under protocol (LA2021334). All experiments were performed under the principle of experimental animal care (NIH publication No. 85Y23, revised 1996) and were approved by the Laboratory Animal Ethics Committee of Peking University (LA2023460).

*Kif13b* knockout (Kif13b^−/−^) and *Kif13b*-floxed (Kif13b^fl/fl^) mice were generated using CRISPR/Cas9 in the Institute of Laboratory Animal Science, Chinese Academy of Medical Sciences, and Peking Union Medical College (Beijing, China). The sgRNAs targeting the sites flanking exon 6 were listed in Additional file 1: Table S1. The donor template was constructed based on the mouse genomic sequence (GRCm39) by inserting two loxP sites on both sides of exon 6. Zygotes microinjection was performed with a mixture of Cas9 protein and sgRNAs with/without a donor template. The primers used for the genotyping of Kif13b^−/−^ and Kif13b^fl/fl^ mice are listed in Additional file 1: Table S1. The liver-specific knockout mice (Kif13b^LKO^) were produced by cross-Kif13b^fl/fl^ mice with Albumin-Cre transgenic mice. As described previously, the *LDLR* knockout (LDLR^−/−^) Syrian golden hamsters were generated using CRISPR/Cas9 [25]. In the experiments described in this article, we used 103 WT mice, 40 Kif13b^−/−^ mice, 18 Kif13b^fl/fl^ mice, 17 Kif13b^LKO^ mice, 6 ob/ob mice, 5 LDLR^−/−^ mice, 5 LDLR^−/−^Kif13b^−/−^ mice; 16 WT hamsters and 17 LDLR^−/−^ hamsters.

The special diets used for the mice in this study were purchased from Research Diet, Inc.: high-fat diet (HFD, D12492), Western diet (WD, D12108C), and methionine-choline-deficient diet (MCD, A02082002BR). For the hamster study, a regular CD containing 20% protein and 4% fat was purchased from Beijing Keao Xieli Feed Co., Ltd., Beijing, China. A high-cholesterol diet (HCD) containing 0.05% and a high-fat and high-cholesterol diet (HFHCD) containing 15% lard and 0.5% cholesterol based on a powdered CD were provided by BiotechHD Co., Ltd., Beijing, China.

The shRNA-adeno-associated virus type 8 (AAV8) sequences used in this experiment are shown in Additional file 1: Table S2. The dose of AAV8 administered via the tail vein was 2 × 10^11^ vg per mouse. The lentivirus was administered in situ via the hepatic portal vein at a dose of 1 × 10^9^ IU. Mice and hamsters were age-matched before being randomly assigned to groups.

### Genotyping

F0 founder animals were identified by PCR followed by sequence analysis and bred with WT mice to generate germline transmission F1 founders. F1 founders were genotyped using tail genomic PCR/DNA sequencing, and a Southern blot examination was performed to further confirm the correct genotype. Hepatic-specific Kif13b^−/−^ mice were identified using One-Step-Mouse Genotyping Kit (PD101-01, Vazyme, China). DNA was extracted from ears of WT and LDLR^−/−^ hamsters using proteinase K (1245680100, MERCK, USA) and tissue lysis buffer, followed by genotyping through PCR. The genotype identification primer sequences are presented in Additional file 1: Table S3 for reference.

### Histology

For histological analysis, the heart, liver, and aorta were harvested and stored at − 80 °C or fixed in 4% paraformaldehyde overnight, followed by dehydration in a 20% sucrose solution. Each liver specimen was evaluated by scanning at low-power, before detailed examination in 5 medium-power fields (20× objective). An experienced pathologist graded MAFLD changes (MAFLD activity score: 0–8) using a modified version of the system described by Kleiner et al. [26]. The following parameters were assessed semi-quantitatively: steatosis (0–3), lobular inflammation (0–3), hepatocyte ballooning degeneration (0–2), and fibrosis grade (0–4). The MAFLD activity score is defined as the sum of the histological scores for steatosis, lobular inflammation, and hepatocyte ballooning degeneration. Each score was calculated as displayed in Additional file 1: Table S4.

#### H&E and Sirius red staining

Paraffin-embedded tissue samples were sectioned, dewaxed, and dehydrated. H&E staining was performed with Harry’s hematoxylin (HHS128-4L; Sigma, Germany) for 5 min and with aqueous eosin (HT110232-1L; Sigma, Germany) for 3 min. For Sirius red staining, sections were stained with Sirius red in saturated picric acid for 15 min.

#### Oil red O (ORO) staining

Frozen samples embedded in optimal cutting temperature compound (4583, Sakura, USA) were sectioned at 7 μm, washed with 60% isopropanol for 10 s, and then stained with 0.3% ORO for 30 min, followed by counterstaining with hematoxylin for 5 min.

#### Immunofluorescence staining

Frozen liver sections were fixed with 4% paraformaldehyde for 30 min. For permeability, the sections were followed by incubation with phosphate buffer saline (PBS) containing 0.1% (v/v) Triton X-100 for 15 min. After blocking in PBS with 3% (w/v) bovine serum albumin (BSA), CD68 (BM3639, Boster, USA), and α smooth muscle actin (α-SMA, BM0002, Boster, USA) antibodies were incubated overnight at 4 °C in the blocking buffer. Afterwards, the sections were washed with PBS containing 0.1% (v/v) Tween 20 and then incubated with Alexa Fluor-conjugated secondary antibodies (Invitrogen, USA) for 1 h at 37 °C in darkness at 1:1000 dilution. 4',6-diamidino-2-phenylindole (DAPI, C1005, Beyotime, China) or Bodipy (D3922, Invitrogen, USA) were also used to display nuclei and lipid droplets, respectively.

#### Image analysis

H&E, Sirius red, or ORO staining were analyzed by Leica DM3000 upright light microscope (Leica, Germany). The slides were scanned using Grundium Ocus^®^ microscope slide scanners (Tampere, Finland) for presentative pictures. The calculation of intensities and positive area was processed by Image-Pro Plus 6.0 software (Media Cybernetics, USA) for quantitation. Super-resolution fluorescent imaging was conducted by All-in-One Fluorescence Microscope BZ-X810 (KEYENCE, USA) with BZ-H4A/Advanced Analysis Software. Image analysis and quantification were performed using Fiji software (9.0).

### Statistical analyses

All statistical tests were performed using GraphPad Prism 9.0 software. All data were presented as means ± standard error of mean (SEM). The normality of the distribution of examined parameters was evaluated using the Shapiro–Wilk test. Differences between the two groups were compared using an unpaired Student’s *t*-test. To compare multiple groups with one- or two-way ANOVA, Tukey’s post hoc analysis was used. Values of *P* < 0.05 were considered statistically significant.

## Results

### Reduced hepatic KIF13B expression in MAFLD patients and mice

**Fig. 1:**
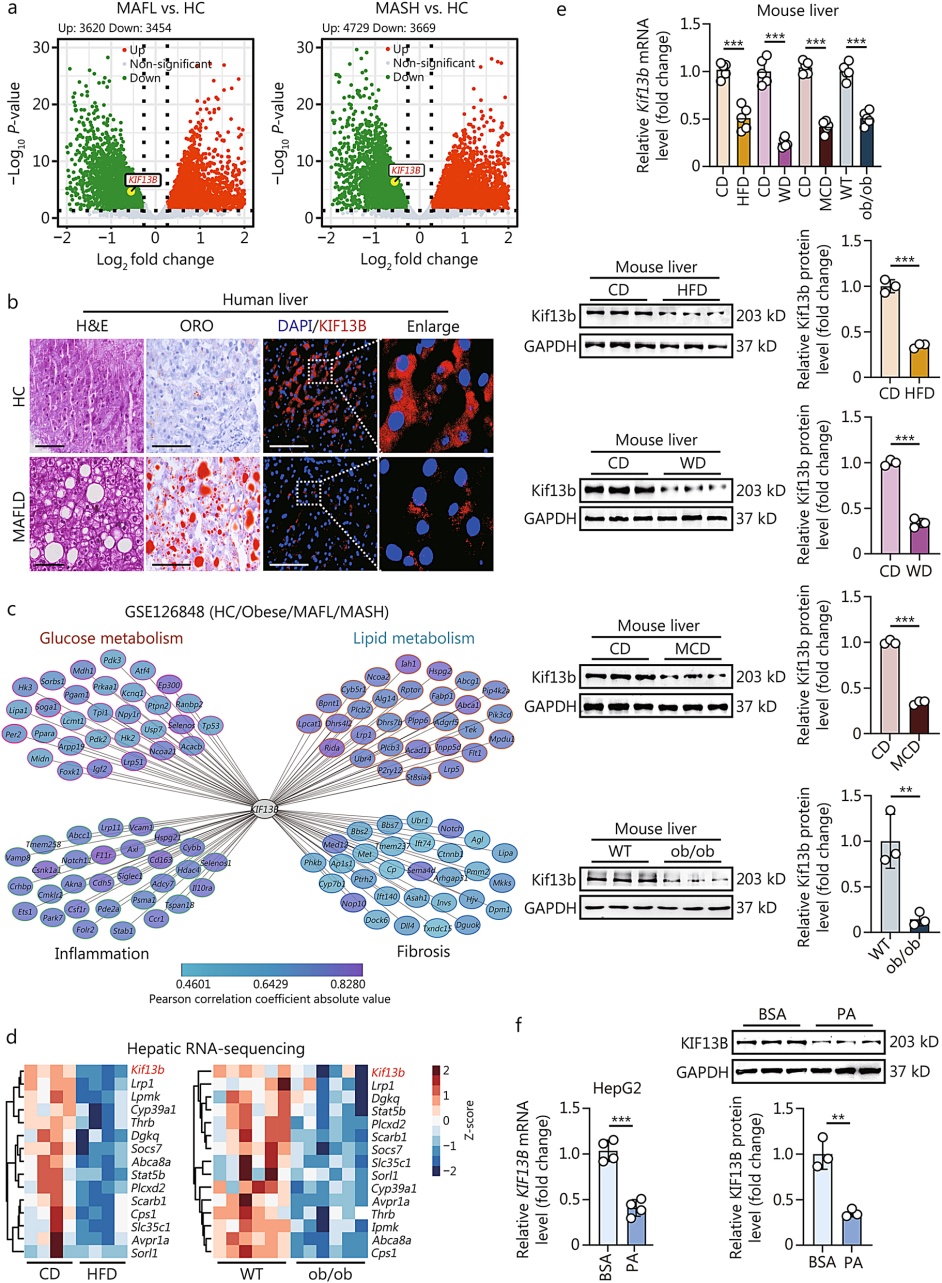
Reduced hepatic KIF13B expression in MAFLD patients and mice.** a** Volcano plot of hepatic *KIF13B* mRNA expressions in metabolic dysfunction-associated fatty liver (MAFL) vs. healthy controls (HC), and metabolic dysfunction-associated steatohepatitis (MASH) vs. HC from Gene Expression Omnibus (GEO) database (GSE126848). **b** Representative images of H&E, oil red O (ORO), and immunofluorescence staining of human KIF13B in liver tissues from HC (*n* = 3) and patients with metabolic dysfunction-associated fatty liver disease (MAFLD,* n* = 18) are presented. The red color in immunofluorescence staining represents the KIF13B protein. Scale bars = 100 μm. **c** Pearson correlation analysis of *KIF13B* expression related to glucose metabolism, lipid metabolism, inflammation, and fibrosis from the same dataset in (**a**). **d** Heat map showing the expression of hepatic *Kif13b* and other genes regulating lipid metabolism in wild-type (WT) mice fed on chow diet (CD) and high-fat diet (HFD) (*n* = 4), as well as WT and ob/ob mice (*n* = 6). **e** The hepatic mRNA and protein levels of *Kif13b* were analyzed in male WT mice on different diets, including a CD, a HFD, a Western diet (WD), a methionine-choline-deficient diet (MCD), and male ob/ob mice (*n* = 5). **f** After starvation for 12 h, HepG2 cells were treated with 0.2% bovine serum albumin (BSA) and palmitic acid (PA; 300 μmol/L) for 24 or 48 h. The mRNA and protein levels of *KIF13B* in the HepG2 cell line were measured. Data are means ± SEM. **P < 0.01, ***P < 0.001. *P*-values were calculated by unpaired two-tailed Student’s *t*-test. KIF13B kinesin family member 13B

To assess the role of KIF13B in MAFLD, we first identified *KIF13B* gene expression in the liver tissues of MAFLD patients from the Gene Expression Omnibus (GEO) datasets and revealed lower *KIF13B* mRNA expression in the livers of patients with MAFL and MASH compared with healthy controls (HC) (Fig. 1a). Likewise, immunofluorescence analysis confirmed the decreased KIF13B protein in the livers of MAFLD patients relative to HC (Fig. 1b). The ensuing correlation analysis showed that KIF13B was closely related to glucose and lipid metabolism, inflammation, and fibrosis in MAFLD (Fig. 1c). Subsequently, we performed mRNA-sequencing on livers of WT mice fed on CD or a HFD, as well as ob/ob mice, a genetic mouse model with spontaneous MAFLD. Consistently, *Kif13b* mRNA expression was dramatically decreased in HFD-fed mice and ob/ob mice compared with their corresponding controls (Fig. 1d). Moreover, both mRNA and protein levels of hepatic *Kif13b* were significantly reduced in different mouse models with MAFLD compared with their control groups (Fig. 1e). Furthermore, in vitro data from HepG2 cell line also demonstrated reduced *KIF13B* mRNA and protein levels upon the treatment of palmitic acid (PA) (Fig. 1f). Overall, these results strongly strengthen the negative association between hepatic KIF13B and MAFLD.

### *Kif13b* deficiency elicits hepatic steatosis and worsens diet-induced steatohepatitis

**Fig. 2:**
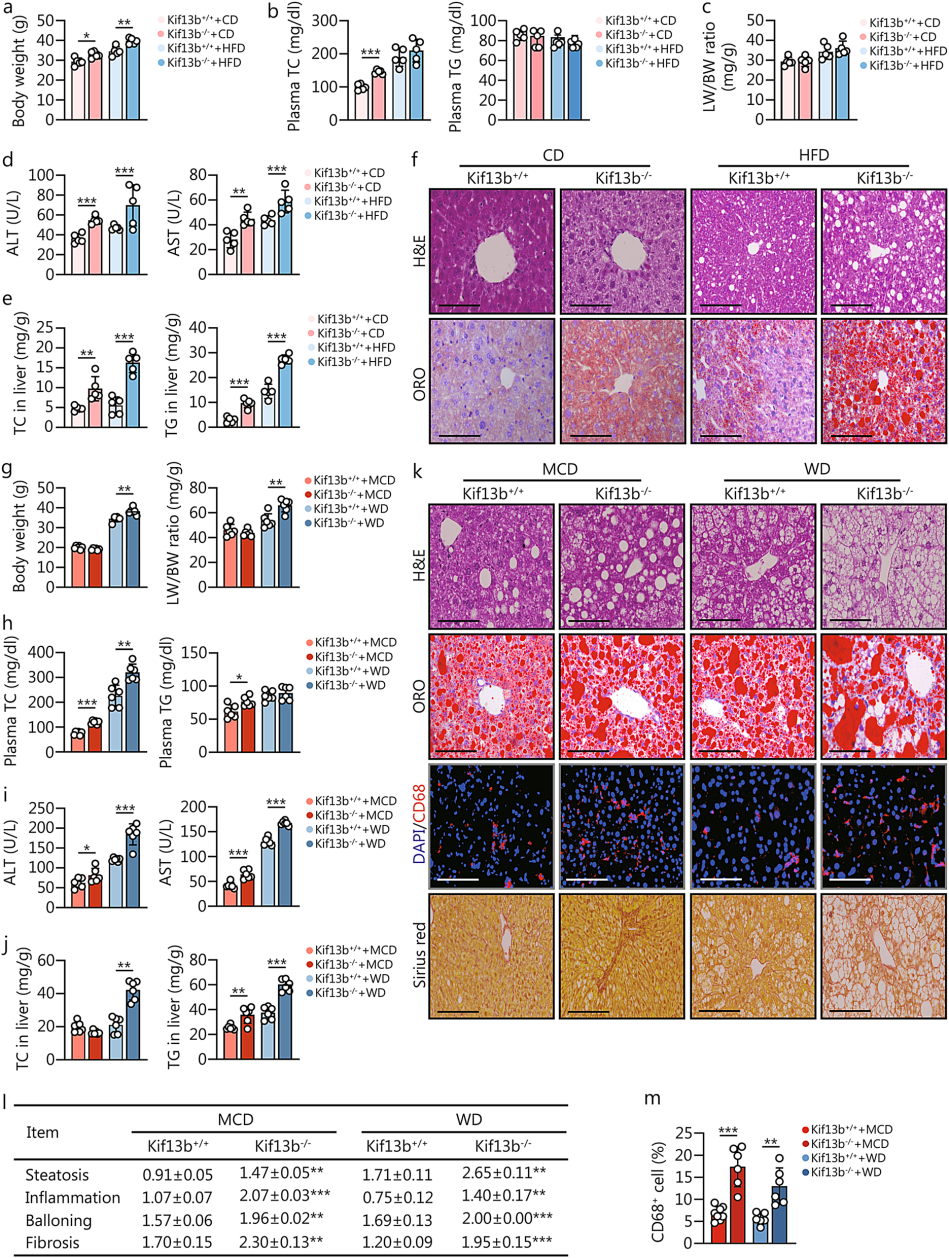
*Kif13b* deficiency elicits hepatic steatosis and worsens diet-induced steatohepatitis. Eight-week-old male Kif13b^+/+^ and Kif13b^−/−^ mice were randomly assigned to two dietary groups: a 16-week chow diet (CD) group for spontaneously hepatic steatosis and an 8-week high-fat diet (HFD) group for diet-induced hepatic steatosis. **a** Body weight of mice fed with CD or HFD. **b** Plasma total cholesterol (TC) and triglyceride (TG) of mice fed with CD or HFD. **c** The ratio of liver weight to body weight (LW/BW) of mice fed with CD or HFD. **d** Plasma alanine aminotransferase (ALT) and aspartate aminotransferase (AST) levels of mice fed with CD or HFD. **e** Liver TC and TG contents of mice fed with CD or HFD. **f** Representative histological images of liver cryo-sections with H&E staining and oil red O (ORO) staining from Kif13b^+/+^ and Kif13b^−/−^ mice fed with CD or HFD. Scale bars = 100 μm. *n* = 5. Eight-week-old male Kif13b^+/+^ and Kif13b^−/−^ mice were randomly assigned to two dietary groups: a 4-week methionine-choline-deficient diet (MCD) group and a 20-week Western diet (WD) group. **g** Body weight and LW/BW ratio of mice fed with MCD or WD. **h** Plasma TC and TG of mice fed with MCD or WD. **i** Plasma ALT and AST levels of mice fed with MCD or WD. **j** Liver TC and TG contents of mice fed with MCD or WD. **k** Representative histological images of liver cryo-sections with H&E staining, ORO staining, immunofluorescence staining of CD68, and Sirius red staining from Kif13b^+/+^ and Kif13b^−/−^ mice fed with MCD or WD. Scale bars = 100 μm. **l** Histological scoring of steatosis, lobular inflammation, hepatocyte ballooning, and fibrosis were calculated. **P < 0.01, ***P < 0.001, Kif13b^−/−^ vs. Kif13b^+/+^. **m** CD68^+^ cell ratio was calculated. *n* = 6. Data are means ± SEM. *P < 0.05, **P < 0.01, ***P < 0.001. *P-*values were calculated by unpaired two-tailed Student’s *t*-test. KIF13B kinesin family member 13B

To explore Kif13b’s impact on MAFLD, we generated a global Kif13b^−/−^ mouse model (Additional file 1: Fig. S1a), and both the genotyping and qPCR using liver tissue confirmed that the *Kif13b* gene had been completely deleted from mice, respectively (Additional file 1: Fig. S1b, c). To better understand the influence of Kif13b on MAFLD, 8-week-old male mice were fed 16-week CD for spontaneous hepatic steatosis or 8-week HFD for diet-induced hepatic steatosis. We first measured the body weight of Kif13b^−/−^ mice and their WT (Kif13b^+/+^) littermates and found that *Kif13b* deficiency caused an increase in body weight under both CD and HFD (Fig. 2a). In compared with WT mice, the plasma total cholesterol (TC) level of CD-fed Kif13b^−/−^ mice was significantly elevated, while the difference was not observed between the two genotypes under an HFD condition (Fig. 2b). In contrast, the plasma triglyceride (TG) level remained unaltered in Kif13b^+/+^ and Kif13b^−/−^ mice fed with either CD or HFD (Fig. 2b). Although the liver-to-body weight (LW/BW) ratio of Kif13b^−/−^ mice did not change significantly compared with control group (Fig. 2c), plasma levels of alanine aminotransferase (ALT) and aspartate aminotransferase (AST) (Fig. 2d), 2 liver injury indicators, and liver TC and TG contents (Fig. 2e) were significantly increased. Further pathological analysis revealed that *Kif13b* deficiency resulted in the spontaneous accumulation of lipids in the liver, which was exacerbated by HFD (Fig. 2f).

To assess whether *Kif13b* ablation could exacerbate the progression of MASH and fibrosis, mice were subjected to a MCD or a WD challenge. For the MCD diet, despite no changes in body weight, the LW/BW ratio, and hepatic TC content, Kif13b^−/−^ mice exhibited elevated plasma TC, TG, ALT, and AST levels (Fig. 2g–j). A significant increase in the content of TG was observed in the livers of Kif13b^−/−^ mice, accompanied by more severe pathological steatosis, inflammation, and fibrosis when compared with control group (Fig. 2j–m).

Under WD conditions, metabolic cage analysis revealed no differences in food intake or ambulatory counts (Additional file 1: Fig. S2a, b). However, Kif13b^−/−^ mice exhibited lower oxygen consumption (VO_2_), carbon dioxide production (VCO_2_), and energy expenditure (EE) than WT mice, with an increased respiratory quotient (RQ) in the dark (Additional file 1: Fig. S2c-f). Magnetic resonance imaging (MRI) analysis showed larger body fat volume and increased liver fat density in Kif13b^−/−^ mice (Additional file 1: Fig. S2g, h), resulting in higher body weight and the LW/BW ratio (Fig. 2g). Plasma TC, ALT, and AST levels were significantly elevated in Kif13b^−/−^ mice, while plasma TG remained unaffected compared with WT mice (Fig. 2h, i). A significant increase in the contents of both TC and TG was observed in the livers of Kif13b^−/−^ mice (Fig. 2j). Histological data confirmed severe lipid accumulation in the livers of Kif13b^−/−^ mice, with more CD68^+^ cells, indicating increased inflammatory cell infiltration (Fig. 2k–m). Additionally, analysis of liver fibrosis using Sirius red staining revealed a significant increase in fibrosis in Kif13b^−/−^ mice as well (Fig. 2k, l). In addition, electron microscopy indicated decreased total number and surface area of mitochondria in Kif13b^−/−^ mice (Additional file 1: Fig. S2i).

### Targeting hepatic Kif13b leads to MASH and fibrosis in mice and hamsters under overnutrient conditions

**Fig. 3:**
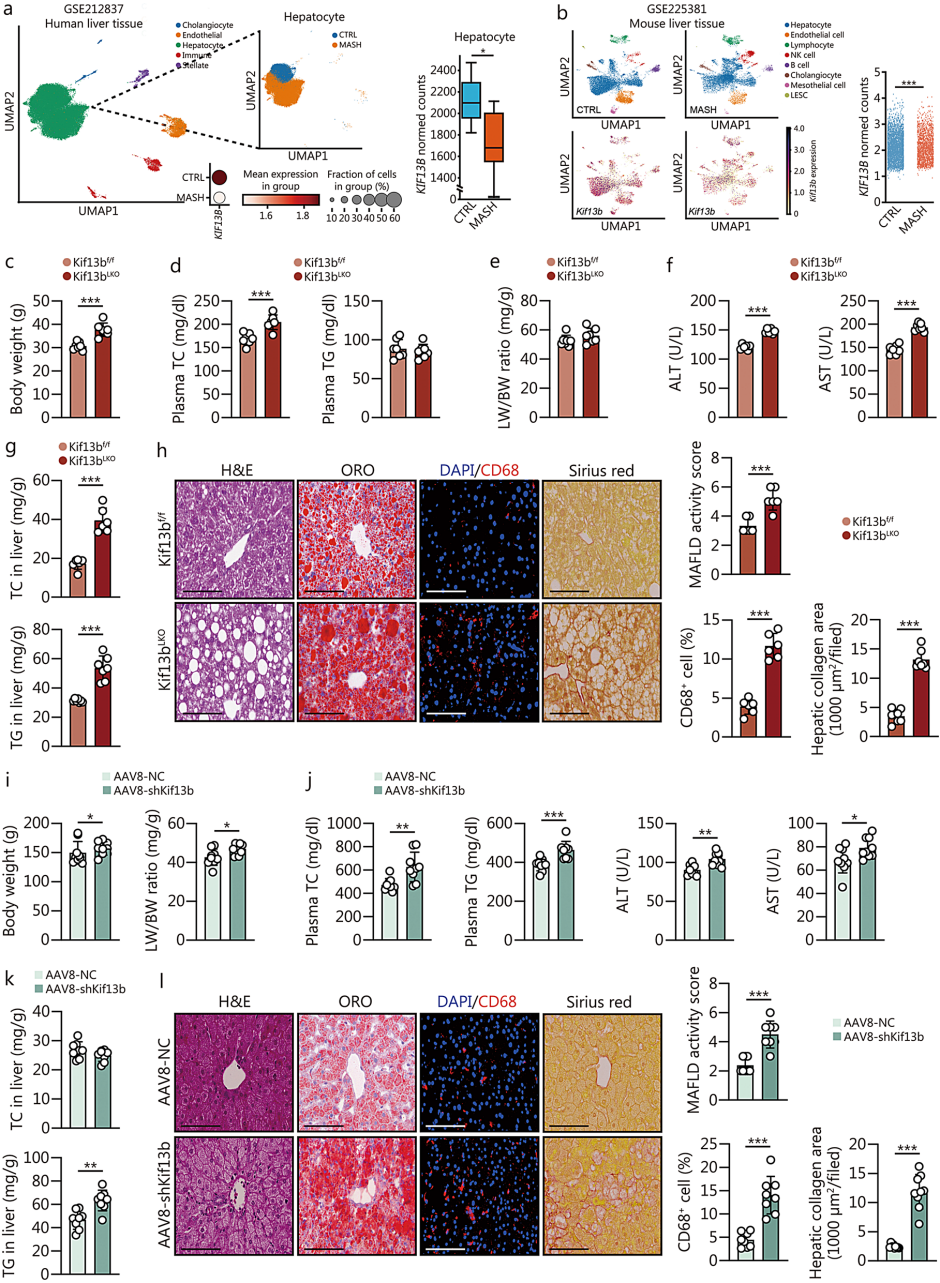
Targeting hepatic *Kif13b* leads to metabolic dysfunction-associated steatohepatitis (MASH) and fibrosis in mice and hamsters under overnutrient conditions. **a** Analysis of single-nucleus RNA sequencing of *KIF13B* mRNA in hepatocytes in human control liver and MASH liver from the Gene Expression Omnibus (GEO) database (GSE212837).** b** Analysis of single-cell RNA sequencing of *Kif13b* mRNA in hepatocytes in mouse control liver and MASH liver from the GEO database (GSE225381). *P-*values were calculated by Deseq2 (Wald’s test) in (**a**) and the one-sided Mann–Whitney *U* test in (**b**). Eight-week-old male Kif13b^f/f^ and Kif13b^LKO^ mice were fed with a Western diet (WD) for 20 weeks. **c** Body weight of indicated mice. **d** Plasma total cholesterol (TC) and triglyceride (TG). **e** The ratio of LW/BW. **f** Plasma alanine aminotransferase (ALT) and aspartate aminotransferase (AST) levels. **g** Liver TC and TG contents. **h** Representative histological images of liver cryo-sections with H&E staining, oil red O (ORO) staining, immunofluorescence staining of CD68, and Sirius red staining from Kif13b^f/f^ and Kif13b^LKO^ mice fed with WD. Scale bars = 100 μm. Histological scoring of MAFLD activity score, CD68^+^ cell ratio, and hepatic collagen areas were presented also. *n* = 6. Eight-week-old WT hamsters received jugular vein injections of AAV8-shKif13b to deplete *Kif13b* from their livers, with AAV8-negative control (NC) as a control. All hamsters were then placed on a CD for 4 weeks, followed by a high-fat and high-cholesterol diet (HFHCD) for another 4 weeks. **i** Body weight and the ratio of LW/BW. **j** Plasma TC, TG, ALT, and AST levels. **k** Liver TC and TG contents. **l** Representative histological images of liver cryo-sections with H&E staining, ORO staining, immunofluorescence staining of CD68, and Sirius red staining. Scale bars = 100 μm. MAFLD activity score, CD68^+^ cells, and hepatic collagen areas were calculated. *n* = 8. Data are means ± SEM. *P < 0.05, **P < 0.01, ***P < 0.001. *P-*values were calculated by unpaired two-tailed Student’s *t* test. KIF13B kinesin family member 13B, MAFLD metabolic dysfunction-associated fatty liver disease

KIF13B was found to be highly expressed in the liver, as evidenced by a previous study [20]. Further analysis of liver tissue from patients and mice with MAFLD using single-nucleus/cell RNA sequencing revealed a significantly reduced *KIF13B* mRNA level in hepatocytes as the disease progressed (Fig. 3a, b). Consequently, we generated Kif13b^LKO^ mice for further study, in which a loss of Kif13b levels was confirmed in the liver and other tissues (Additional file 1: Fig. S3a-d). Strikingly, lack of hepatic *Kif13b* led to increased body weight and elevated circulating TC and ALT levels in CD-fed mice, without significant changes in LW/BW ratio and plasma TG and AST levels (Additional file 1: Fig. S3e-h). Meanwhile, Kif13b^LKO^ mice exhibited increased hepatic TC and TG contents and spontaneous accumulation of lipids in the liver (Additional file 1: Fig. S3i, j).

Notably, metabolic cage data revealed that Kif13b^LKO^ mice displayed higher food intake, VO_2_, and EE under CD condition, while other parameters such as VCO_2_, RQ, and ambulatory counts remained unchanged (Additional file 1: Fig. S4a–f), which might lead to obesity and spontaneously hepatic steatosis in Kif13b^LKO^ mice fed CD for 16 weeks. In conclusion, these data indicate that targeting hepatic Kif13b is sufficient to disrupt liver metabolic homeostasis without dietary intervention.

Upon WD feeding, Kif13b^LKO^ mice also displayed increased body weight and elevated plasma TC, ALT, and AST levels; however, plasma TG level and LW/BW ratio showed no alteration (Fig. 3c–f). Additionally, WD-fed Kif13b^LKO^ mice exhibited more hepatic TC and TG contents and serious steatosis, inflammation, and fibrosis (Fig. 3g, h), mirroring the findings observed in Kif13b^−/−^ mice.

In this context, hepatic *Kif13b* silencing was induced in WT hamsters using AAV8-shKif13b, followed by a HFHCD to explore Kif13b’s role in the pathogenesis of MAFLD (Additional file 1: Fig. S5a). qPCR data showed that *Kif13b* mRNA was effectively silenced by 95% in hamster livers (Additional file 1: Fig. S5b). Hamsters lacking hepatic *Kif13b* exhibited increased body weight and LW/BW ratio, elevated levels of plasma TC, TG, ALT, and AST compared with hamsters injected with AAV8-negative control (NC) (Fig. 3i, j), accompanied with more liver TG content (Fig. 3k). Histological analysis also confirmed more extensive lipid accumulation, macrophage infiltration, and fibrosis in the livers of hamsters with hepatic *Kif13b* deficiency compared with control hamsters (Fig. 3l). Collectively, these data indicated that *Kif13b* deficiency in hepatocytes is sufficient to cause lipid metabolism disorders and to exacerbate MASH and fibrosis in hamsters.

### Hepatic KIF13B overexpression prevents MASH in MCD-fed mice

To verify the protective effect of hepatocyte KIF13B on MASH and fibrosis, a liver-specific human KIF13B overexpression mouse model was established by hepatic portal vein injection of lentivirus vector expressing KIF13B (LV-KIF13B), and the mice were fed an MCD diet for 3 weeks. LV-KIF13B injection significantly increased liver *Kif13b* at both mRNA and protein levels in WT mice (the sequence identity between human and mouse *KIF13B* mRNA was 86%, while the amino acid sequence identity was 88%. Consequently, the sequence shared by human and mouse was employed to design primers and antibodies) (Additional file 1: Fig. S6a, b). *KIF13B* overexpression did not alter the body weight but decreased the LW/BW ratio (Additional file 1: Fig. S6c, d). Moreover, LV-KIF13B-infected mice displayed a reduced level of circulating TC, ALT, and AST without affecting plasma TG level (Additional file 1: Fig. S6e). Consistently, decreased hepatic TC and TG contents and less macrophage infiltration and hepatic stellate cell activation were observed in *KIF13B* overexpression mice (Additional file 1: Fig. S6f, g). In parallel, *KIF13B* overexpression lowered mRNA expression levels of fatty acid synthesis (*Fasn*), inflammation markers (*Il1β*, *Tnfα*, *Ifnγ, Il10*, and *Mcp1*), fibrosis markers (*αsma*), and increased lipid export (*Abcg1* and *Mttp*) (Additional file 1: Fig. S6h, i).

### Depletion of ***Kif13b*** predisposes LDLR^−/−^ mice and hamsters to atherosclerosis

Given the known association between MAFLD and atherosclerotic cardiovascular disease (ASCVD) [27], the present study investigated the potential causal link between *Kif13b* deficiency and atherosclerosis in the context of MAFLD. We crossed Kif13b^−/−^ mice with LDLR^−/−^ mice to generate a double mutant mouse model lacking both *Kif13b* and *LDLR*. Intriguingly, *Kif13b* ablation did not influence body weight but resulted in increased plasma TC, TG, non-high-density lipoprotein cholesterol (non-HDLC), and malondialdehyde (MDA) levels in WD-fed LDLR^−/−^ mice (Additional file 1: Fig. S7a, b). Analysis of plasma lipid profiles by fast protein liquid chromatography (FPLC) revealed higher TC and TG contents in chylomicron/very-low-density lipoprotein (CM/VLDL) and LDL fractions in LDLR^−/−^Kif13b^−/−^ mice when compared with the LDLR^−/−^ group (Additional file 1: Fig. S7c), with the former showing more extensive aortic lesions in the entire aorta (Additional file 1: Fig. S7d). Moreover, since no *Kif13b* knockout hamsters have been established, we applied AAV8-shKif13b to silence liver *Kif13b* in LDLR^−/−^ hamsters (Additional file 1: Fig. S7e). Interestingly, although deletion of *Kif13b* from the liver of LDLR^−/−^ hamsters had no noticeable effect on body weight and plasma TC and non-HDLC levels, it indeed led to elevated circulating TG and MDA levels (Additional file 1: Fig. S7f, g). FPLC analysis revealed higher TG contents in CM/VLDL and LDL fractions, while TC contents remained unchanged in different fractions in LDLR^−/−^ hamsters injected with AAV8-shKif13b when compared with the LDLR^−/−^ hamsters injected with AAV8-NC group (Additional file 1: Fig. S7h). Correspondingly, aortic lesions were markedly increased in the LDLR^−/−^ hamsters lacking hepatic *Kif13b* (Additional file 1: Fig. S7i). Altogether, these findings suggest that Kif13b may represent a potential therapeutic target for the treatment of atherosclerosis in the presence of MAFLD.

### Kif13b regulates lipid synthesis and mitochondrial function

**Fig. 4:**
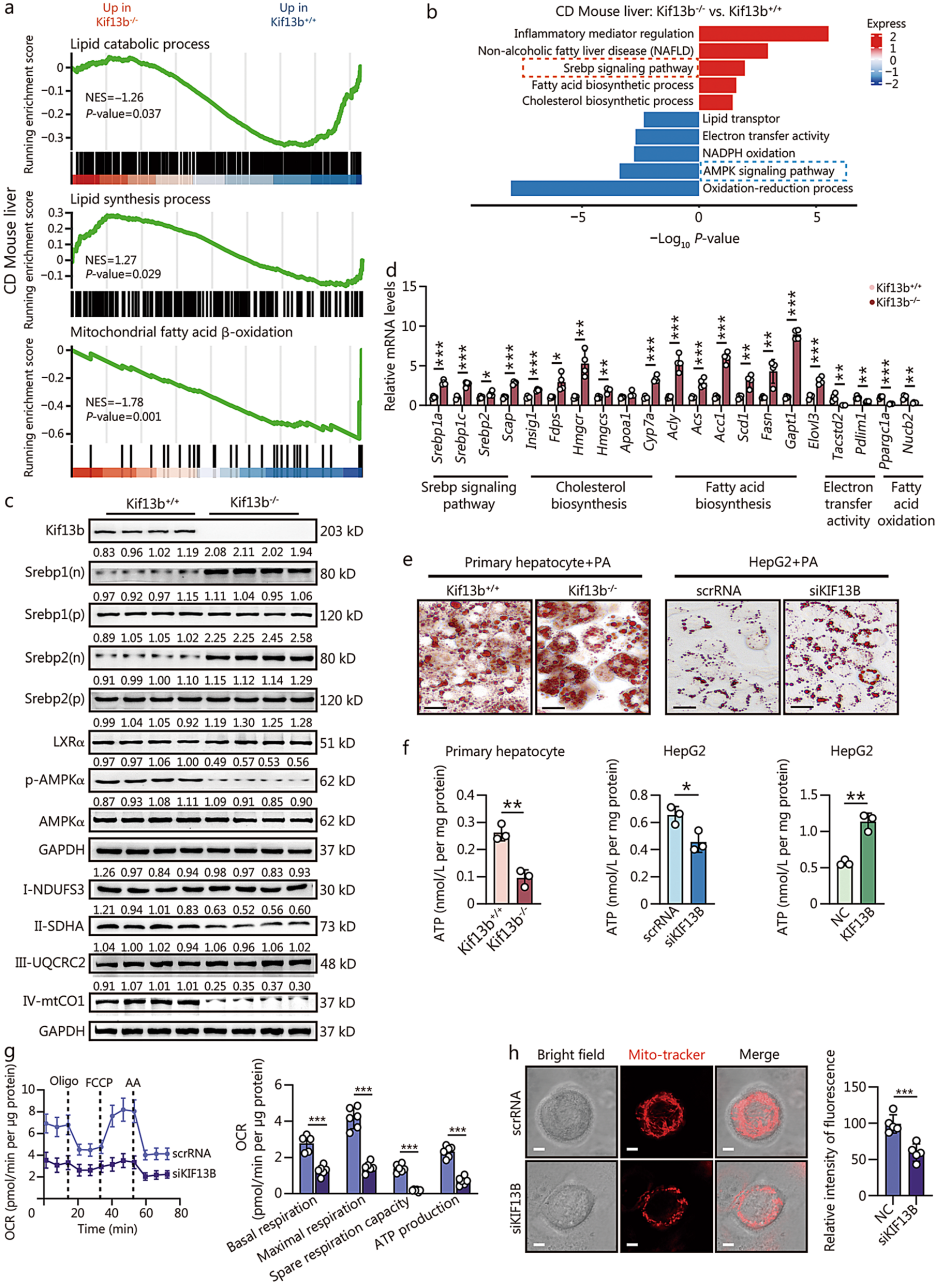
Kif13b regulates lipid synthesis and mitochondrial function. Gene set enrichment analysis (GSEA) (**a**) and Kyoto Encyclopedia of Genes and Genomes (KEGG) (**b**) analysis based on the RNA sequencing in the livers of male Kif13b^+/+^ and Kif13b^−/−^ mice fed with chow diet (CD) (*n* = 5). **c** Western blotting analysis of liver samples from CD-fed mice. **d** The mRNA expression profiles of genes associated with the Srebp signalling pathway, cholesterol biosynthesis, fatty acid biosynthesis, electron transport activity, and fatty acid oxidation in the liver of mice fed a CD. **e** Oil red O staining of primary mouse hepatocytes and HepG2 cells following *KIF13B* silencing by siRNA exposure to palmitic acid (PA; 300 μmol/L) after 12 h of starvation. Scale bars = 100 μm. **f** ATP production of primary mouse hepatocytes and HepG2 cells with *KIF13B* silence by siRNA or *KIF13B* overexpression by plasmid exposure with PA (300 μmol/L) after 12 h of starvation. **g** Oxygen consumption rates (OCR) were measured by Seahorse XF96 analyzer in *KIF13B* knockdown HepG2 cells (*n* = 6). OCR relating to mitochondrial basal respiration, maximal respiration, spare respiration capacity, and ATP production were respectively analyzed and normalized to the corresponding total protein content per well in *KIF13B* knockdown HepG2 cells. **h** Representative images demonstrating alterations in the morphology and number of mitochondria in HepG2 cells following *KIF13B* silencing were captured using a confocal microscope. Scale bars = 2 μm. The relative fluorescence quantification of mitochondria was shown also. Data are means ± SEM. *P < 0.05, **P < 0.01, ***P < 0.001. *P-*values were calculated by unpaired two-tailed Student’s* t*-test. KIF13B kinesin family member 13B, NES normalized enrichment score, Srebp sterol regulatory element binding protein

To explore the molecular mechanism by which Kif13b governed MAFLD progression, we analyzed mRNA sequencing data from the livers of CD- or HFD-fed Kif13b^+/+^ and Kif13b^−/−^ mice. A total of 5784 and 1810 differential expression genes (DEGs) were measured, and 194 overlapped DEGs were used as input for further Gene Ontology (GO) analysis, which indicated that *Kif13b* deficiency significantly alters lipid metabolism and mitochondrial biogenesis and regulation in the liver (Additional file 1: Fig. S8a, b). Gene set enrichment analysis (GSEA) demonstrated that *Kif13b* deletion resulted in the upregulation of genes regulating lipid synthesis processes and the downregulation of genes related to mitochondrial fatty acid β-oxidation processes (Fig. 4a). Kyoto Encyclopedia of Genes and Genomes (KEGG) analysis revealed that the Srebp signaling pathway was significantly enriched in Kif13b^−/−^ mice, while the AMPK signaling pathway was inhibited, 2 signaling pathways that are closely associated with lipid synthesis and mitochondrial metabolism (Fig. 4b). Western blotting showed elevated levels of nuclear Srebp1 and Srebp2 in Kif13b^−/−^ mouse livers, alongside with reduced AMPKα phosphorylation (Fig. 4c). In agreement, we also observed reduced levels of Complex II-SDHA and IV-mtCO1 proteins in the livers of Kif13b^−/−^ mice, indicating the impairment of electron transport chain (Fig. 4c). Concurrently, the mRNA levels of factors related to Srebp signaling pathway, cholesterol and fatty acid biosynthesis in the livers of Kif13b^−/−^ mice were significantly elevated, while the mRNA levels of factors related to electron transfer activity and fatty acid oxidation were markedly decreased (Fig. 4d). In vitro experiments consistently showed increased lipid droplet accumulation and reduced ATP production in primary mouse hepatocytes lacking *Kif13b*, as well as in HepG2 cells with *KIF13B* silence by siRNA (Fig. 4e, f). Conversely, overexpression of human *KIF13B* increased ATP production in HepG2 cells (Fig. 4f). Besides, mitochondrial oxygen consumption rates (OCR) relating to basal respiration, maximal respiration, spare respiration capacity, and ATP production were markedly blunted with *KIF13B* knockdown in HepG2 cells (Fig. 4g), accompanied by decreased mitochondrial abundance (Fig. 4h).

### Kif13b orchestrates lipid metabolism and mitochondrial function through AMPKα

**Fig. 5:**
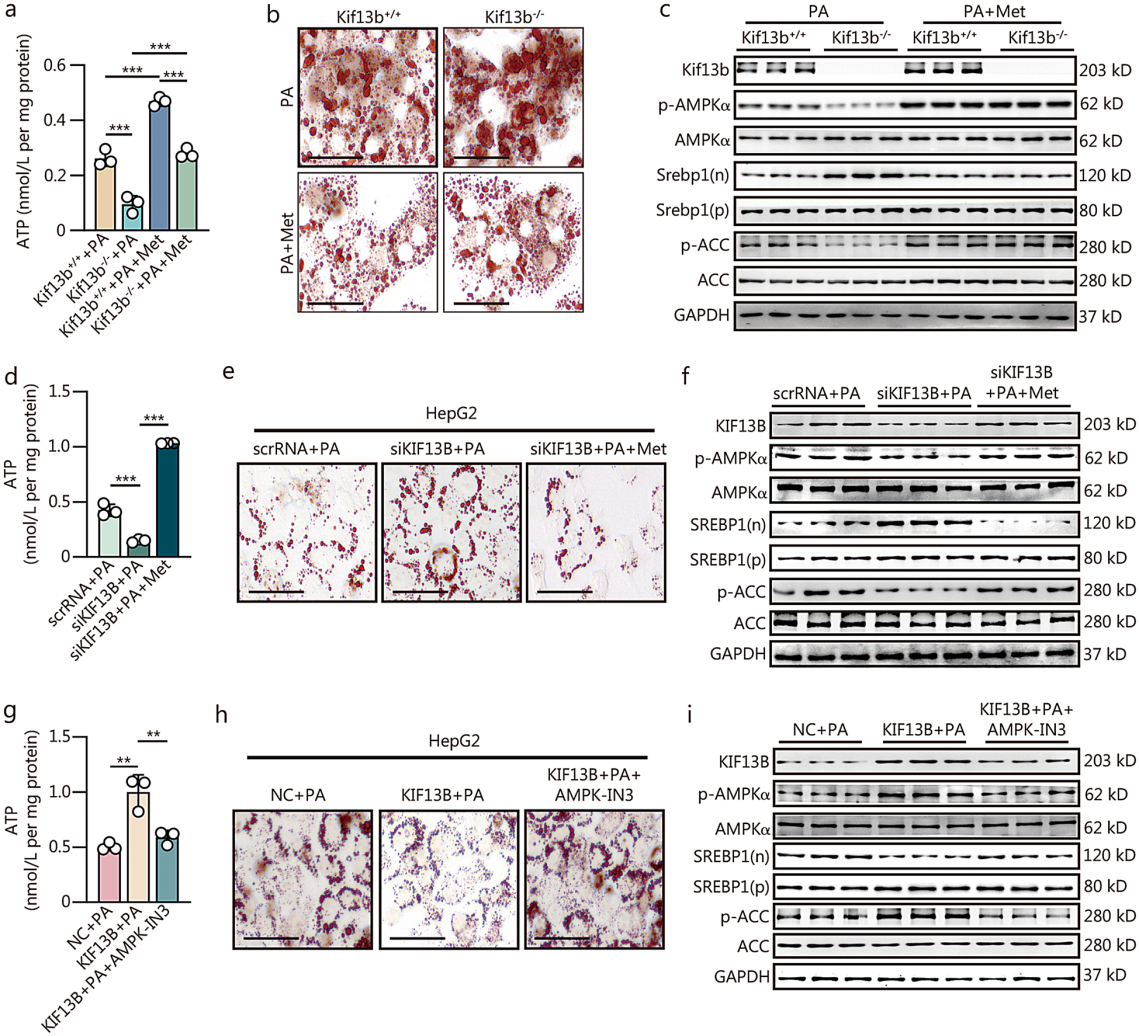
Kif13b orchestrates lipid metabolism and mitochondrial function in an AMPKα dependent manner. Primary mouse hepatocytes from Kif13b^+/+^ and Kif13b^−/−^ mice were starved 12 h and then exposed to palmitic acid (PA) at a concentration of 300 μmol/L with or without metformin (Met) at 2 mmol/L for 24 h. **a** ATP production in primary mouse hepatocytes. **b** Representative images from oil red O (ORO) staining. **c** Representative images of Western blotting. In HepG2 cells, transfection with scrambled RNA (scrRNA) or siKIF13B occurred for 48 h, followed by exposure to 300 μmol/L PA and 2 mmol/L Met for a further 24 h, after 12 h of starvation. **d** ATP production in HepG2 cells. **e** Representative images from ORO staining. **f** Representative images of Western blotting. HepG2 cells were transfected with a negative control (NC) or KIF13B plasmid for 48 h and treated with 300 μmol/L PA and 110 nmol/L AMPK-IN3 (an AMPKα inhibitor) for a further 24 h, after 12 h of starvation. **g** ATP production in HepG2 cells. **h** Representative images from ORO staining. **i** Representative images of Western blotting. Each experiment was independently replicated three times. Scale bars = 100 μm. Data are means ± SEM. **P < 0.01, ***P < 0.001. *P-*values were calculated by one- or two-way ANOVA followed by Tukey’s test. KIF13B kinesin family member 13B

Subsequently, we conducted in vitro experiments to explore whether Kif13b integrates lipid metabolism and mitochondrial function in hepatocytes by manipulating AMPKα activity. Primary mouse hepatocytes were cultured in PA-containing media with or without metformin, an AMPKα activator. Hepatocytes lacking *Kif13b* exhibited decreased ATP production, increased lipid accumulation, reduced AMPKα phosphorylation, and elevated nuclear Srebp1 level and acetyl-CoA carboxylase (ACC) phosphorylation, all of which were significantly reversed by the treatment with metformin (Fig. 5a–c, Additional file 1: Fig. S9a). Similar results were obtained in *KIF13B*-silenced HepG2 cells, where metformin effectively reversed the impact of KIF13B deficiency on lipid accumulation and SREBP1 nuclear translocation (Fig. 5d–f, Additional file 1: Fig. S9b). Conversely, overexpression of human *KIF13B* had a beneficial effect on these parameters, which was counteracted by an AMPKα inhibitor (AMPK-IN3) (Fig. 5g–i, Additional file 1: Fig. S9c). In conclusion, the findings indicate that AMPKα activation is required for the beneficial effects of KIF13B on lipid metabolism and mitochondrial function to maintain hepatic metabolic homeostasis in vitro.

### Metformin improves *Kif13b* knockout-induced MAFLD by activating AMPKα

**Fig. 6:**
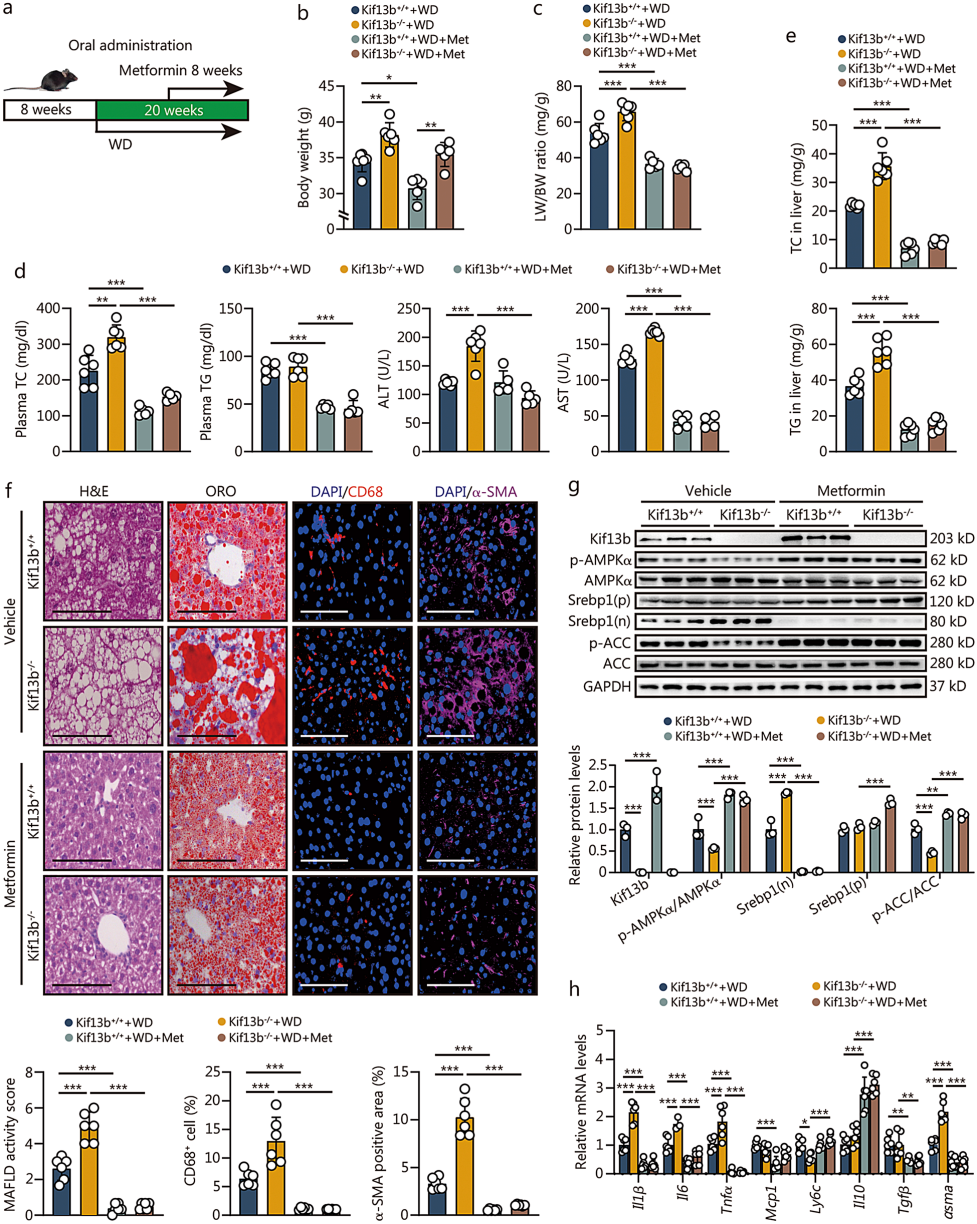
Metformin improves *Kif13b* deficiency-induced MAFLD by activating AMPKα. **a** Eight-week-old male mice were subjected to a 20-week Western diet (WD) and received vehicle or metformin every 2 d via gavage for 8 weeks. **b** Body weight. **c** The ratio of liver weight to body weight (LW/BW). **d** Plasma total cholesterol (TC), triglyceride (TG), alanine aminotransferase (ALT), and aspartate aminotransferase (AST) levels. **e** Liver TC and TG contents. **f** Representative histological images of liver cryo-sections with H&E staining, oil red O (ORO) staining, and immunofluorescence staining of CD68 and α smooth muscle actin (α-SMA) from mice in (**a**). Scale bars = 100 μm. Histological scoring of MAFLD activity score, CD68^+^ cells ratio and α-SMA positive area were calculated. **g** Representative images and relative quantification of Western blotting of liver samples from mice in (**a**). **h** mRNA levels of proinflammatory cytokines and fibrosis markers of liver samples from mice in (**a**). *n* = 6. Data are means ± SEM. *P < 0.05, **P < 0.01, ***P < 0.001. *P-*values were calculated using two-way ANOVA followed by Tukey’s test. KIF13B kinesin family member 13B, MAFLD metabolic dysfunction-associated fatty liver disease

Next, we evaluated the beneficial effect of AMPKα activation on MAFLD in the context of *Kif13b* deficiency in vivo. Kif13b^+/+^ and Kif13b^−/−^ mice were maintained on WD for 20 weeks and then administered metformin through gavage every 2 d for 8 weeks (Fig. 6a). Metformin treatment was found to significantly reduce body weight, LW/BW ratio, and plasma levels of TC, TG, and AST in Kif13b^+/+^ mice when compared with Kif13b^+/+^ mice receiving vehicle (Fig. 6b–d). Importantly, the elevation of LW/BW ratio and plasma TC, ALT, and AST levels due to *Kif13b* deficiency was also rescued by metformin (Fig. 6c, d). In addition, metformin was effective to reduce the contents of TC and TG in the livers of both Kif13b^+/+^ and Kif13b^−/−^ mice (Fig. 6e). Consistently, metformin treatment abrogated lipid deposition, macrophage infiltration, and hepatic stellate cell activation (Fig. 6f). Mechanistically, metformin treatment increased AMPKα and ACC phosphorylation and decreased nuclear Srebp1 levels in the livers of both Kif13b^+/+^ and Kif13b^−/−^ mice (Fig. 6g). This, in turn, suppressed the expression of genes associated with lipogenesis, inflammation, and fibrosis, ultimately preventing WD-induced MAFLD (Fig. 6h, Additional file 1: Fig. S10). Taken together, these results show that *Kif13b* deficiency exacerbates WD-induced MAFLD, which can be reversed by metformin through the activation of AMPKα.

### AMPKα1 deficiency inhibits the protective effect of KIF13B on MAFLD

**Fig. 7:**
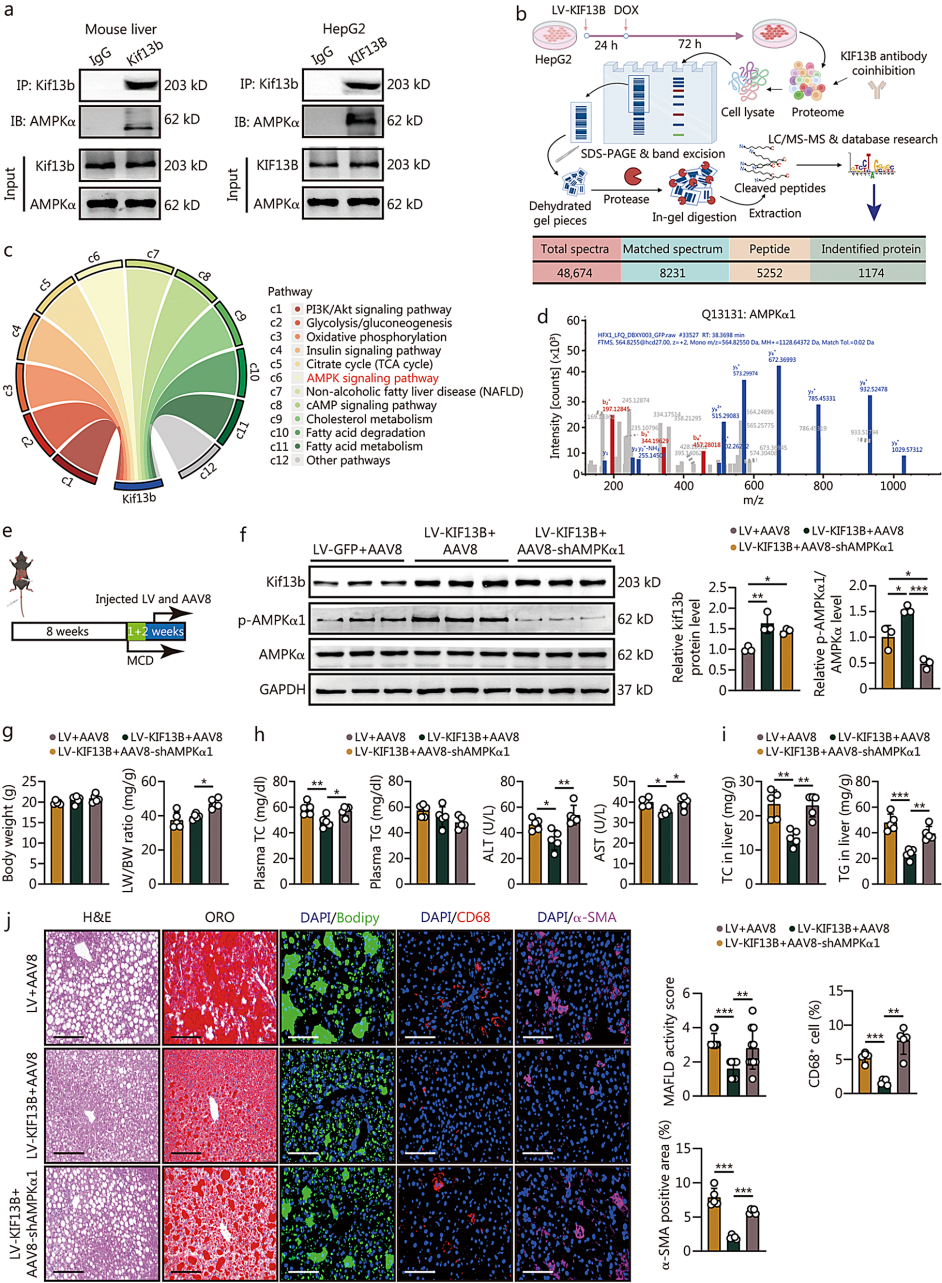
AMPKα1 deficiency inhibits the protective effect of KIF13B on MAFLD. **a** Co-immunoprecipitation (Co-IP) experiments investigate the interaction between Kif13b and AMPKα in WT mouse livers and HepG2 cells overexpressing KIF13B. **b** Following the overexpression of KIF13B-GFP in HepG2 cells, liquid chromatography-tandem mass spectrometry (LC–MS/MS) analysis of purified proteins co-immunoprecipitated with Kif13b-GFP employed GFP protein as a control, and the 1174 identified protein was observed exclusively in the Kif13b-GFP group. **c** Kyoto Encyclopedia of Genes and Genomes (KEGG) pathway enrichment analysis based on the proteins identified in (**b**). Pathways with *P* < 0.05 were chosen and sorted by -log_10_ *P*-value in reverse order. **d** Protein secondary structure analysis of AMPKα1. **e** Eight-week-old male WT mice were subjected to a 1-week diet of methionine-choline-deficient (MCD) feeding. In addition to lentivirus (LV) or LV-KIF13B through the portal vein, they were administered either AAV8 or AAV8-shAMPKα1 via tail vein injection, followed by another 2-week MCD diet. **f** Western blotting analysis of liver samples from mice in (**e**). Relative quantification of Kif13b to GAPDH and the ratio of phosphorylated AMPKα1 to total AMPKα. **g** Body weight and the ratio of liver weight to body weight (LW/BW). **h** Plasma total cholesterol (TC), triglyceride (TG), alanine aminotransferase (ALT), and aspartate aminotransferase (AST) levels. **i** Liver TC and TG contents. **j** Representative histological images of liver cryo-sections with H&E staining, oil red O (ORO) staining, and immunofluorescence staining of bodipy, CD68, and α smooth muscle actin (α-SMA) from mice in (**e**). Scale bars = 100 μm. Histological scoring of MAFLD activity score, CD68^+^ cells ratio and α-SMA positive area were calculated. *n* = 5. Data are means ± SEM. *P < 0.05, **P < 0.01, ***P < 0.001. *P-*values were calculated by one-way ANOVA followed by Tukey’s test. KIF13B kinesin family member 13B, MAFLD metabolic dysfunction-associated fatty liver disease, AMPKα1 AMP-activated catalytic subunit alpha 1

Based on the observations above, we verified that the phenotypes of Kif13b^−/−^ mice were linked to suppressed liver AMPKα activity. Co-immunoprecipitation (Co-IP) experiments further confirmed an interaction between Kif13b and AMPKα in WT mouse liver and HepG2 cells overexpressing KIF13B (Fig. 7a). A comprehensive investigation conducted over the past decade has uncovered a multitude of AMPKα isoforms exhibiting distinct tissue expression patterns [28]. Consequently, to identify which AMPKα isoforms participate in the role of KIF13B in MAFLD, we conducted an overexpression of KIF13B-GFP in HepG2 cells, and then used the protein lysates pulled down by a GFP antibody to detect interactome by liquid chromatography-tandem mass spectrometry (LC–MS/MS). The results demonstrated that KIF13B could interact with a total of 1174 proteins, which were primarily involved in glucose and lipid metabolism and mitochondrial homeostasis (Fig. 7b). Furthermore, these proteins were significantly enriched in the AMPK signaling pathway (Fig. 7c). Protein secondary structure analysis revealed that KIF13B is specifically bound to AMPKα1 (Fig. 7d). We performed a transfection study in HepG2 cells to investigate the effect of KIF13B and its different functional domains on AMPKα1 phosphorylation and lipid accumulation. Immunoblotting results showed that full-length KIF13B and its CAP-Gly domain, the only functional domain of KIF13B, significantly bound with AMPKα1 and enhanced the phosphorylation of AMPKα1 (Additional file 1: Fig. S11a, b). ORO staining showed that overexpression of KIF13B or the CAP-Gly domain significantly reduced PA-induced lipid accumulation in HepG2 cells (Additional file 1: Fig. S11c). These results suggest that the KIF13B CAP-Gly functional domain plays an important role in the regulation of AMPKα1 activation and lipid metabolism. To further ascertain the potential involvement of KIF13B in the progression of MAFLD through AMPKα1, WT mice were subjected to an MCD diet for 1 week, after which they were divided into LV + AAV8, LV-KIF13B + AAV8, LV-KIF13B + AAV8 + shAMPKα1 groups, as illustrated in Fig. 7e. All mice were then maintained on an MCD diet for an additional 2 weeks. Western blotting data revealed a significant increase in Kif13b protein levels with LV-mediated overexpression and a marked reduction in AMPKα1 protein phosphorylation levels with AAV8-mediated knockdown (Fig. 7f). Notably, there were no differences in body weight and plasma TG level among the three groups (Fig. 7g, h). Although *Kif13b* overexpression did not affect the LW/BW ratio, it did lead to reduced plasma TC, ALT, and AST levels compared with control group (Fig. 7g, h). In contrast, mice with *AMPKα1* knockdown exhibited an increased LW/BW ratio, along with elevated plasma TC, ALT, and AST levels compared with LV-KIF13B-treated mice (Fig. 7g, h). When compared with control mice, LV-KIF13B-treated mice displayed lower hepatic TC and TG contents (Fig. 7i), and improved lipid accumulation, inflammatory cell infiltration, and stellate cell activation in the livers (Fig. 7j); however, AAV8-shAMPKα1 counteracted the protective effects of KIF13B overexpression on liver damage (Fig. 7i, j), demonstrating that AMPKα1 is a key molecule responsible for the protection of Kif13b against MAFLD.

## Discussion

**Fig. 8:**
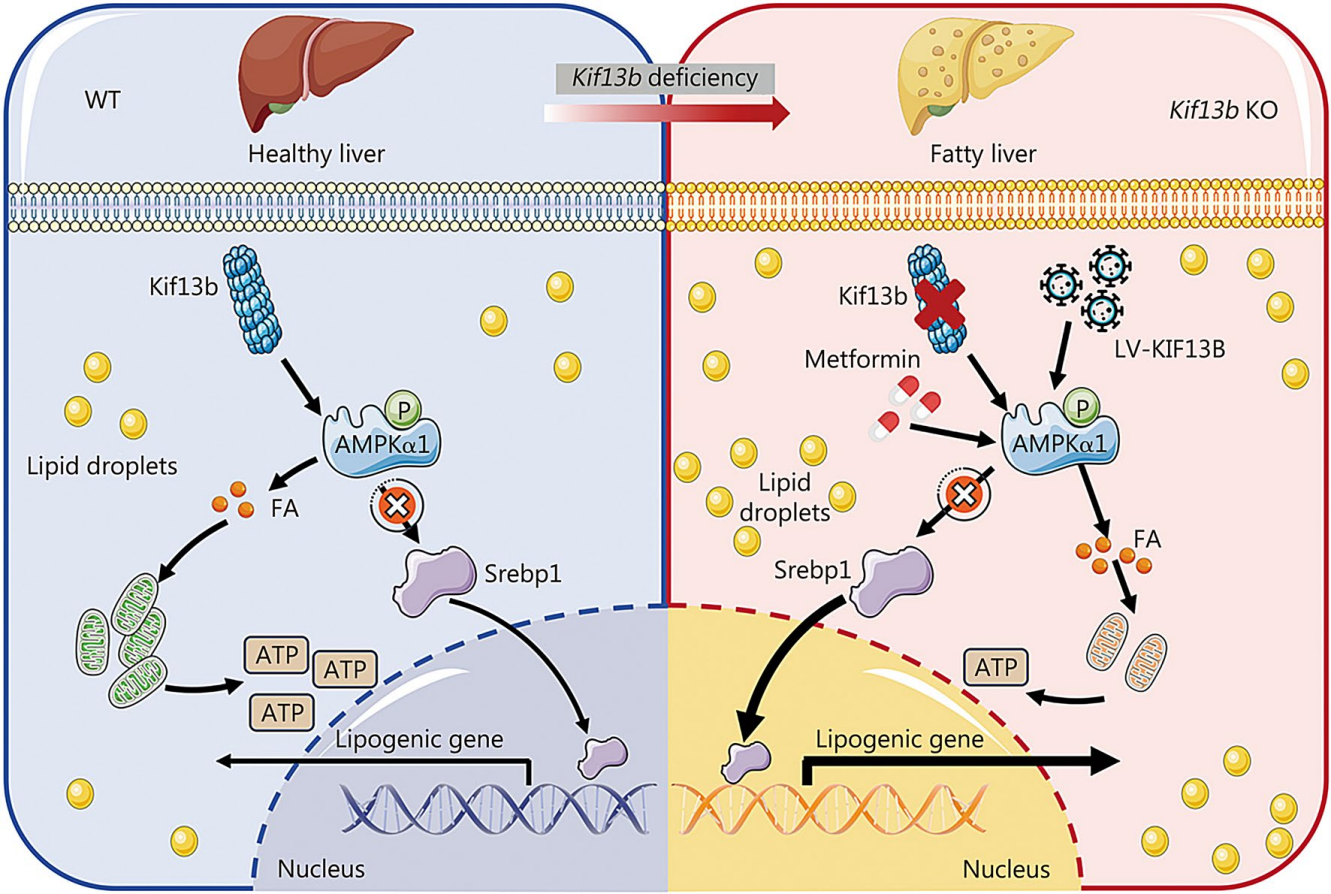
Deletion of *Kif13b* in the liver exacerbates MASH through suppression of AMPKα activity. In physiological settings, Kif13b plays a crucial role in enhancing AMPKα activity, inhibiting Srebp1 nuclear translocation, fostering fatty acid oxidation, and preserving mitochondrial homeostasis. Nonetheless, in the liver’s absence of Kif13b, the phosphorylation of AMPKα is impeded, resulting in a disturbance in lipid metabolism and mitochondrial function. Consequently, this disturbance contributes to the accumulation of excess lipids, thereby intensifying the progression of MASH. Conversely, the restoration of AMPKα phosphorylation through either KIF13B overexpression or the administration of metformin proves effective in mitigating MASH. KIF13B kinesin family member 13B, AMPKα1 AMP-activated catalytic subunit alpha 1, WT wild-type, FA fatty acid, KO knockout, MASH metabolic dysfunction-associated steatohepatitis, Srebp1 sterol regulatory element binding protein 1

Despite significant strides in understanding MAFLD pathogenesis, identifying therapeutic targets, and advancing drug development, the field still grapples with unmet clinical challenges. To date, thyroid hormone receptor-β agonist has been approved by the FDA to treat MASH; however, it still is urgent to identify new potential therapeutic targets because the pathogenesis underlying MAFLD is too complex and one drug is not sufficient to treat this disorder with broad spectrum [29–31]. In the present study, we found reduced KIF13B levels in patients and murine models with MAFLD. It has been reported that *KIF13B* DNA may be subject to modification by methylation [32], which has the potential to downregulate KIF13B expression. Accordingly, we conducted a comparative analysis of the KIF13B methylation profile at three sites (cg02835742, cg18875839, and cg23731836) in the liver samples from healthy and MAFLD patients. Our findings revealed that these three KIF13B promoter CpGs exhibited increased methylation in the livers of MAFLD patients relative to HC tissues, which probably explained why *KIF13B* mRNA and protein expression levels were significantly reduced in the livers of MAFLD patients. Nevertheless, the potential mechanism underlying KIF13B methylation remains uncertain and warrants precise investigation in future studies. Rodents with global or liver-specific knockout of the *Kif13b* gene exhibited spontaneous hepatic steatosis, which was further exacerbated by different overnutrition diets. We also revealed that overexpression of human *KIF13B* by LV effectively prevented MASH in MCD-fed mice. Furthermore, *Kif13b* deficiency accelerated atherosclerosis in the context of MAFLD. Mechanistically, *Kif13b* depletion increased hepatic lipid synthesis and impaired mitochondrial oxidative phosphorylation. Further screening revealed that Kif13b interacted with AMPKα1 to regulate the phosphorylation of AMPKα1, governing mitochondrial homeostasis and suppressing Srebp1-mediated DNL in the liver. Therefore, this work establishes a causal relationship between *Kif13b* deficiency and MAFLD, emphasizing Kif13b as a potential therapeutic target for treating MAFLD (Fig. 8).

As the previous study has shown, KIF13B interacts with centaurin-α1, a protein that regulates mitochondrial function, in neurons [17], indicating that KIF13B might be involved in the regulation of mitochondrial function. It has been reported that mitochondria homeostasis plays a central role in MAFLD, with patients often displaying mitochondrial dysfunction characterized by varying degrees of mitochondrial ultrastructural damage, abnormal morphological changes, decreased respiratory chain activity, ATP depletion, imbalanced mitochondrial functions, including biogenesis, fission, fusion and autophagy, impaired mitochondrial β-oxidation, increased permeability of outer and inner membranes, ROS overproduction, and oxidative stress-mediated mtDNA loss [5, 33–35]. Kif13b possesses ATPase activity that converts the chemical energy of ATP into mechanical work and then enables to regulate multiple cellular processes, such as intracellular cargo long-range transport and organelle division [17, 18], prompting an inquiry into its potential implications in the context of MAFLD.

MAFLD is a condition of fat accumulation in the liver in combination with metabolic dysfunction. Hepatic lipid metabolism is regulated by a combination of the uptake and export of fatty acids, DNL, and fat utilization by β-oxidation. When the balance between these pathways is disrupted, hepatic lipid accumulation commences, and long-term activation of inflammatory and fibrotic pathways can progress to worsen liver disease [36]. Intriguingly, transcriptomic profiling of the liver tissue indicated that loss of Kif13b enhanced DNL mediated by Srebps, inhibited the AMPK signaling pathway and impaired the mitochondrial function in mice on both CD and HFD feeding conditions. Of note, AMPK-mediated signaling transduction is widely appreciated for its multiple profound impacts on the pathogenesis underlying MAFLD, primarily through four principal mechanisms: (1) attenuation of DNL in hepatocytes by inhibiting the maturation, cleavage and translocation of Srebp1 and Srebp2 and enhancing ACC phosphorylation, (2) augmentation of hepatic fatty acid oxidation via increasing CPT1 expression, (3) promotion of mitochondrial function and integrity, and (4) modifying the assembly of VLDL to promote hepatic TG efflux [37–41]. Interestingly, we discovered that Kif13b’s role mirrored AMPK-mediated signaling, indicating that Kif13b, beyond its conventional role as a motor protein responsible for integrating mitochondrial movement and ATPase activity, may serve as an upstream modulator of AMPK activation. In this capacity, Kif13b could wield influence over mitochondria by orchestrating AMPK phosphorylation events, thereby participating in protective measures against MAFLD. Notably, the Co-IP experiment revealed an interaction between Kif13b and AMPKα, and silencing *Kif13b* resulted in a diminishment of AMPKα phosphorylation. Consequently, this perturbation of AMPKα phosphorylation due to *Kif13b* deficiency led to increased lipid synthesis, decreased fatty acid oxidation, and mitochondrial dysfunction in hepatocytes, which ultimately caused lipid accumulation and ATP production reduction. Encouragingly, the introduction of metformin, a commercially available AMPKα activator, proved efficacious in reversing this proclivity. As AMPKα is comprised of various isoforms and plays a pivotal role in the pathogenesis of MAFLD [42, 43], to ascertain whether KIF13B regulates AMPKα as a single subunit or as a complex, we conducted LC–MS/MS following the enrichment of KIF13B in vitro and demonstrated that KIF13B specifically binds to AMPKα1. To further substantiate the influences of KIF13B on MAFLD via AMPKα1, our in vivo experiments furnished additional evidence, demonstrating that elevated KIF13B expression effectively alleviated MASH pathogenesis induced by an MCD. Nevertheless, this protective effect was abrogated when AMPKα1 was selectively silenced, which confirmed that AMPKα1 played an essential role in the protection from MAFLD in the setting of KIF13B overexpression. Our observations were consistent with the previous findings showing that the deficiency of AMPKα1 exacerbated hepatic lipid accumulation in mice subjected to an HFD, while metformin inhibited an HFD-induced fatty liver by activating AMPKα1 [43]. Furthermore, the application of metformin manifested the capacity to ameliorate various MAFLD-related phenotypes in both WT and Kif13b^−/−^ mice, thereby underscoring its potential to mitigate the deleterious consequences of *Kif13b* deficiency in the context of MAFLD. This study has demonstrated that Kif13b can protect MAFLD by activating AMPKα. It should be noted that AMPK activators, such as A-769662, PF-739, or metformin, have been shown to ameliorate the symptoms of MAFLD-hepatic steatosis, inflammation, liver injury, and fibrosis via different mechanisms [44]; however, global activation of AMPK by MK-8722 has been observed to result in cardiomyocyte hypertrophy, possibly due to the induction of cardiac glycogen synthesis [45]. Given that increased AMPK activity results in some side effects, such as cardiomyocyte hypertrophy, liver-specific activation of AMPK by liver cell-targeted drug delivery might be of great interest for the treatment of MASH. Consequently, the potential use of AMPK as a target for clinical MAFLD therapy requires further verification in future clinical trials. Importantly, unlike other previous reports showing that Kif13b’s phosphorylation by protein kinases influences its microtubule binding and cargo transport capabilities [46, 47], in the present study, we identified a novel interaction between Kif13b and AMPKα1, which in turn impacted AMPKα1 phosphorylation but not expression, suggesting that Kif13b per se could serve as a key molecule to control the enzymatic activity of AMPKα1 by affecting its phosphorylation. However, the specific role of Kif13b in this process remains unclear. Further experiments will be warranted to elucidate whether Kif13b may play a role as a serine-threonine kinase to phosphorylate AMPKα1 or act as a scaffold to facilitate AMPKα1 phosphorylation.

Hyperlipidemia with elevated plasma cholesterol and/or TG levels is one of the risk factors of atherosclerosis, characterized by the buildup of plaques due to the excess lipid accumulation in the vascular wall [48–50]. To date, emerging lines of evidence demonstrate that MAFLD increases the incidence of ASCVD, suggesting a causal link between MAFLD and atherosclerosis [51, 52]. Furthermore, the earlier the onset of MAFLD, the higher the risk of cardiovascular disease [53]. It is crucial to reinforce the prevention, screening, and management of ASCVD risk in patients with early-onset MAFLD. We extended to an evaluation of the potential ramifications of Kif13b in atherosclerotic development. To this end, we leveraged LDLR^−/−^ mice and hamsters subjected to a dietary regimen that precipitated MAFLD. Our investigations revealed that Kif13b deficiency generated a more atherogenic lipid profile with elevated plasma levels of TC, TG, and MDA in LDLR^−/−^ mice. To our surprise, no difference in plasma TC was found in LDLR^−/−^ hamsters with or without hepatic Kif13b. This discrepancy could be explained by the possibilities: (1) compared with LDLR^−/−^ mice fed with WD containing 40% fat and 1.25% cholesterol, LDLR^−/−^ hamsters fed with only 0.05% cholesterol showed high sensitivity to diet-induced hyperlipidemia and more severe hypercholesterolemia with plasma TC level higher than 2000 mg/dl, which masked the detrimental effect of *Kif13b* deficiency; (2) although the mRNA expression of hamster *Kif13b* in the liver was undetectable after AAV8-shKif13b delivery, confirming very high efficiency shRNA-based *Kif13b* knockdown, we cannot exclude the unreported compensatory pathways that regulate lipid metabolism, especially cholesterol metabolism, from other tissues with Kif13b expression. Nevertheless, loss of hepatic *Kif13b* consistently resulted in increased TG and MDA levels in circulation, two factors positively associated with ASCVD [54, 55]. As expected, the inactivation of Kif13b promoted atherosclerotic development in both mice and hamsters with familial hypercholesterolemia, a condition where such lesions are prominently featured. Since the process of atherosclerosis entails the participation of diverse cellular constituents, including vascular endothelial cells, vascular smooth muscle cells, macrophages, and so on, the precise influence of Kif13b on these cellular events and its potential modulation of plasma lipid levels necessitates further comprehensive investigation. Concurrently, the relationship between MAFLD and ASCVD encompasses multiple disciplines, including hepatology, cardiology, endocrinology, and nutrition. In the future, it will be necessary to pursue more extensive interdisciplinary collaboration to investigate and resolve this complex health issue jointly.

In conclusion, herein our novel findings report for the first time that KIF13B expression levels are significantly reduced in the livers of patients and multiple murine and hamster models with MAFLD, and deletion of Kif13b from rodent animals, especially liver tissue is sufficient to promote MAFLD, which establish a causal link between reduced liver Kif13b levels and MAFLD and identify Kif13b as a crucial regulator responsible for AMPKα1 activation to uphold mitochondrial homeostasis, mitigate hepatic lipid accumulation, and restrain inflammatory responses, thereby delineating a promising avenue for the treatment and management of MAFLD and its associated ASCVD.

## Conclusions

Herein, our novel findings reveal a reduction in KIF13B levels in MAFLD patients and overnourished murine models. Rodents with global or liver-specific knockout of the *Kif13b* gene exhibit spontaneous hepatic steatosis, which is further exacerbated by different overnutrition diets. We also revealed that overexpression of human KIF13B by lentivirus effectively prevented MASH in MCD-fed mice. Furthermore, *Kif13b* deficiency accelerates atherosclerosis in the context of MAFLD. Mechanistically, *Kif13b* depletion results in increased hepatic lipid synthesis and impaired mitochondrial oxidative phosphorylation. Further screening reveals that Kif13b interacts with AMPKα1 to regulate the phosphorylation of AMPKα1, governing mitochondrial homeostasis and suppressing Srebp1-mediated DNL in the liver. Therefore, this work establishes a causal relationship between KIF13B deficiency and MAFLD, emphasizing KIF13B as a potential therapeutic target for the treatment of MAFLD.

## Supplementary Information

**Additional file 1:** **Methods. Table S1** Sequences of sgRNAs. **Table S2** shRNA sequences. **Table S3** Primer sequences for genotyping. **Table S4** MAFLD activity score. **Table S5** Primary antibodies. **Table S6** Human *KIF13B* gene siRNA sequences. **Table S7** Mouse qPCR primer sequences. **Fig. S1** Construction of a *Kif13b* knockout mouse model. **Fig. S2** *Kif13b* deficiency disrupts energy metabolism in mice on a Western diet (WD). **Fig. S3** Hepatic *Kif13b* deletion elicits hepatic steatosis. **Fig. S4** Energy metabolism in liver-specific *Kif13b*-deficient mice. **Fig. S5** A hepatic Kif13b knockdown hamster model was constructed using adeno-associated virus vector (AAV) 8. **Fig. S6** Hepatic *KIF13B* overexpression prevents metabolic dysfunction-associated steatohepatitis (MASH) in methionine-choline-deficient diet (MCD)-fed mice. **Fig. S7** Depletion of *Kif13b* predisposes LDLR^−/−^ mice and hamsters to atherosclerosis. **Fig. S8** Kif13b regulates lipid synthesis and mitochondrial function in the mouse liver. **Fig. S9** Kif13b orchestrates lipid metabolism and mitochondrial function through AMPKα. **Fig. S10** Metformin improves *Kif13b* knockout-induced liver injury. **Fig. S11** Identification of CAP-Gly domain of Kif13b as the binding site for AMPKα1.

## Data Availability

All data are available in the main text or the Supplementary materials. The publicly available datasets analyzed to assess Kif13b transcription profiles during the current study are available in the Gene Expression Omnibus repository with the accession codes GSE126848, GSE212837, and GSE225381. The datasets generated and analyzed during the current study are available from the corresponding author upon reasonable request.

## Abbreviations

AAV8: Associated virus type 8
ACC: Acetyl-CoA carboxylase
ALT: Alanine aminotransferase
AMPKα: AMP-activated catalytic subunit α
AST: Aspartate aminotransferase
α-SMA: α Smooth muscle actin
ASCVD: Atherosclerotic cardiovascular disease
ATP: Adenosine triphosphate
BSA: Bovine serum albumin
CD: Chow diet
CM/VLDL: Chylomicron/very-low-density lipoprotein
Co-IP: Co-immunoprecipitation
DEGs: Differential expression genes
DNL: De novo lipogenesis
EE: Energy expenditure
ETC: Electron transport chain
FDA: Food and Drug Administration
FPLC: Fast protein liquid chromatography
GEO: Gene expression omnibus
GSEA: Gene set enrichment analysis
GO: Gene Ontology
HCD: High-cholesterol diet
HDL: High-density lipoprotein
HFD: High-fat diet
HFHCD: High-fat and high-cholesterol diet
KEGG: Kyoto encyclopedia of genes and genomes
Kif13b: Kinesin family member 13b
LDL: Low-density lipoprotein
LRP1: LDL receptor related protein 1
LV: Lentivirus
MAFLD: Metabolic dysfunction-associated fatty liver disease
MASH: Metabolic dysfunction-associated steatohepatitis
MCD: Methionine-choline-deficient diet
MDA: Malondialdehyde
MRI: Magnetic resonance imaging
Non-HDLC: Non-high-density lipoprotein cholesterol
RQ: Respiratory quotient
ORO: Oil red O
PBS: Phosphate buffer saline
ROS: Reactive oxygen species
TC: Total cholesterol
TG: Triacylglycerol
VCO_2_: Carbon dioxide production
VO_2_: Oxygen consumption
WD: Western diet
