# Supplementary material for: Motor protein KIF13B orchestrates hepatic metabolism to prevent metabolic dysfunction-associated fatty liver disease
Source: Mil Med Res. 2025 Mar 4;12:11. doi: 10.1186/s40779-025-00594-3 (PMC11877712; doi:10.1186/s40779-025-00594-3)
Supplement: Supplementary file 1 — Additional file 1: Methods. Table S1 Sequences of sgRNAs. Table S2 shRNA sequences. Table S3 Primer sequences for genotyping. Table S4 MAFLD activity score. Table S5 Primary antibodies. Table S6 Human KIF13B gene siRNA sequences. Table S7 Mouse qPCR primer sequences. Fig. S1 Construction of a Kif13b knockout mouse model. Fig. S2 Kif13b deficiency disrupts energy metabolism in mice on a Western diet (WD). Fig. S3 Hepatic Kif13b deletion elicits hepatic steatosis. Fig. S4 Energy metabolism in liver-specific Kif13b-deficient mice. Fig. S5 A hepatic Kif13b knockdown hamster model was constructed using adeno-associated virus vector (AAV) 8. Fig. S6 Hepatic KIF13B overexpression prevents metabolic dysfunction-associated steatohepatitis (MASH) in methionine-choline-deficient diet (MCD)-fed mice. Fig. S7 Depletion of Kif13b predisposes LDLR−/− mice and hamsters to atherosclerosis. Fig. S8 Kif13b regulates lipid synthesis and mitochondrial function in the mouse liver. Fig. S9 Kif13b orchestrates lipid metabolism and mitochondrial function through AMPKα. Fig. S10 Metformin improves Kif13b knockout-induced liver injury. Fig. S11 Identification of CAP-Gly domain of Kif13b as the binding site for AMPKα1. [file 40779_2025_594_MOESM1_ESM.pdf]

Methods

Lipid extraction

Lipids were extracted from approximately 30 mg of frozen tissues using a modified version of Bligh and Dyer's method as described previously [1]. Briefly, tissues were homogenized in 900 µl of chloroform: methanol: MilliQ H_2O (3:6:1; v/v/v). The homogenate was then incubated at 1500 rpm for 30 min at 4 °C. At the end of the incubation, 350 µl of deionized water and 300 µl of chloroform were added to induce phase separation. The samples were then centrifuged and the lower organic phase containing lipids was extracted into a clean tube. Lipid extraction was repeated once by adding 500 µl of chloroform to the remaining aqueous phase, and the lipid extracts were pooled into a single tube and dried in the SpeedVac under OH mode. Samples were stored at –80 °C until further analysis.

Transmission electron microscopy

Mice were anesthetized and transcardially perfused by normal saline and followed by freshly prepared 1% glutaraldehyde (G5882, Sigma, Germany) with 2% paraformaldehyde (P6148, Sigma, Germany) in 0.1 mol/L phosphate buffer (PB), pH 7.4. Fixed tissues were cut into 1 mm^3 blocks and incubated with 2.5% glutaraldehyde with 2% paraformaldehyde in 0.1 mol/L PB (pH 7.4) for 1 h at room temperature, and then stored overnight at 4 °C. After washing several times with 0.1 mol/L PB, the samples were treated with 0.1 mol/L imidazole in 0.1 mol/L PB (pH 7.4) for 1 h at room temperature and post-fixed in 1% OsO_4/0.1 mol/L imidazole (56749, Sigma, Germany) in 0.1 mol/L PB (pH 7.4) for 1 h at room temperature avoiding light. Specimens were washed with distilled water and en bloc stained with 2% aqueous uranyl acetate overnight at 4 °C. Following several washes in distilled water, specimens were dehydrated through a graded ethanol series (30%, 50%, 70%, 85%, 95%, 100%; 8 min each) and exchanged with pure acetone two times for 8 min each. Tissue blocks were gradually permeated and embedded in epoxy resin EMbed 812 (14120, Electron Microscopy Sciences). Polymerized resin blocks were trimmed and sectioned with diamond knives (Diatome, Switzerland) using a Leica Microsystem UC7 ultramicrotome. Ultra-sections (75 nm) were mounted on copper grids with a single slot, counterstained with uranyl acetate and lead citrate and examined under a transmission electron microscope Tecnai G2 Spirit BioTWIN (Thermo Fisher Scientific) equipped with a CCD camera (Orius 832, Gatan) at 120 kV.

Magnetic resonance imaging (MRI) of mouse liver and fat

MRI was performed using a 3.0T Discovery MR750 (GE Healthcare, USA), with a mouse body coil (CG, China). Mice were anesthetized with 1.5% isoflurane (R510-22, RWD, China) in 1 L/min of compressed air. The MRI protocol contains three sequences. First, a T2-weighted rapid acquisition

relaxation enhanced sequence was acquired [repetition time (RT) = 3512 ms, echo time (ET) = 24 ms, number of excitation (NEX) = 2, slice thickness = 2 mm, slices = 19, field of view (FOV) = 10.0 cm × 7.5 cm, matrix = 320 × 320, acquisition time = 2:59 min]. Second, a T2-weighted rapid acquisition relaxation enhanced sequence with water saturation (TR = 3595 ms, TE = 24 ms, NEX = 2, slice thickness = 2 mm, slices = 19, FOV = 10.0 cm × 7.5 cm, matrix = 320 × 320, acquisition time = 3:04 min) was acquired to evaluate the fat distribution. Last, a rapid acquisition relaxation enhanced sequence with Dixon water and fat separation (TR = 3512 ms, TE = 24 ms, NEX = 2, slice thickness = 2 mm, slices =19, FOV = 10 × 7.5 cm^2, matrix = 256 × 256, acquisition time = 7:23 min) was acquired to evaluate the proton density fat faction.

Energy metabolism characterization

To assess systemic energy metabolism, mice were housed in a single cage for 24 h according to the prevention and early reversibility protocols. Subsequently, the different groups of mice were monitored in the Oxymax Lab Animal Monitoring System by experimental designs. The experimental diets and water were available ad libitum. Finally, 24-hour monitoring data were obtained, including calorie intake, locomotion, oxygen consumption, carbon dioxide production, respiratory quotient (RQ), and energy expenditure (EE). Locomotion was reported as total beam breaks of the X and Y axes.

Blood collection

For different study purposes, anticoagulant or non-anticoagulant blood samples were collected from the orbital vein after 4 h (mice) or 12 h (hamsters) fasting. Plasma or serum were separated by centrifugation for 10 min at 4 °C. The samples were stored at –80 °C for future analysis.

Biochemical analysis

Plasma total cholesterol (TC) and triglyceride (TG) concentrations were measured by the commercially available kits from Biosino Bio-Technology & Science Inc (Beijing, China). High-density lipoprotein cholesterol (HDL-C) was determined after ApoB precipitation by PEG3000. Alanine aminotransferase (ALT; C009-2-1, Njjcbio, China) and aspartate aminotransferase (AST; C010-2-1, Njjcbio, China) were also measured by the commercially available kits, respectively, according to the manufacturer’s instructions.

The levels of adenosine triphosphate (ATP) in hepatocyte mitochondria were determined using the ATP luminescence detection assay system (S0027, Beyotime, China) following the manufacturer’s

recommendations. Mitochondrial fractions were obtained using the Mitochondrial Fractionation Kit (C3601, Beyotime, China).

Western blotting

Total proteins from cultured cells or collected tissues were extracted using ice-cold RIPA buffer (P0013B, Beyotime, China), supplemented with a protease inhibitor cocktail and a phosphatase inhibitor cocktail. Protein concentrations in lysates were quantified using a BCA protein assay kit (23225, Thermo Fisher Scientific, USA). The lysates were mixed with loading buffer (P0015L, Beyotime, China) and denaturized at 95 °C for 10 min. Then the samples were analyzed by SDS-PAGE and nitrocellulose membrane transfer. The membranes were blocked with 5% non-fat milk in TBST buffer (25 mmol/L Tris, 137 mmol/L NaCl, 2.7 mmol/L KCl, 0.075% Tween-20) at room temperature for 1 h, and then incubated with the indicated primary antibody in TBST buffer containing 5% bovine serum albumin (BSA) at 4 °C overnight. Afterward, the membranes were rinsed and incubated with horseradish peroxidase-conjugated secondary antibody (Zsgb-Bio, China) in TBST buffer supplemented with 5% BSA at room temperature for 1 h. Pierce ECL Plus Western blotting substrate (32209, Thermo Fisher Scientific, USA) was used to visualize the purpose proteins. Primary antibodies used for immunoblotting are listed in **Additional file 1: Table S5**.

Isolation and culture of primary hepatocytes

Primary hepatocytes were isolated from the livers of 8-week-old male WT (Kif13b$^{+/+}$) or Kif13b$^{-/-}$ mice, as described previously [2]. Briefly, the liver was subjected to perfusion through the inferior vena cava using 0.5 mg/ml type IV collagenase (C5138, Sigma, Germany) after anesthesia. Cell viability, determined using Trypan blue dyes, exceeded 85%. Subsequently, hepatocytes were isolated, and cultured in high glucose DMEM medium with 10% fetal bovine serum, 100 U/ml penicillin, and 0.1 mg/ml streptomycin for 8 h. Following a 12-hour starvation period, primary hepatocytes were exposed to fresh medium, which included palmitic acid (PA, 300 μmol/L, P0500, Sigma, Germany) dissolved with 0.2% BSA, with or without metformin (2 mmol/L, 317240, Sigma, Germany), for an additional 24 h.

Co-immunoprecipitation (Co-IP) assay

Liver tissues or cultured cells were lysed in 1 ml RIPA buffer on ice for 30 min. After vortexing for 15 s, lysates were centrifuged at 4 °C for 30 min at 12,000 r/min. Eighty microliters of supernatants were mixed with 20 μl 5× loading buffer as an input sample. Eight hundred microliters of supernatants were incubated with 10 μl anti-KIF13B antibody at 4 °C for 6 h. Conjugated protein

A/G agarose beads were added overnight at 4 °C. After being washed in lysis buffer 5 times at 4 °C, beads were boiled with 120 μl 1× loading buffer at 95 °C for 10 min and then centrifuged at 12,000 r/min for 5 min. The supernatants were used as pellet samples. Twenty microliters of samples were analyzed by immunoblotting.

Plasmid constructs and transfection

Plasmids were constructed using standard molecular cloning techniques. The coding sequence (excluding the stop codon) of *Kif13b* (NM_015254) was amplified by polymerase chain reaction (PCR) and inserted into the expression vector pcDNA3.1. HepG2 cells (HB-8065, ATCC, USA) were plated in a 6-well plate (3×10^5 cells/well), cultured with Opti-MEM medium, and 2 μg plasmids were transfected with Lipo8000 (C0533, Beyotime, China) according to the manufacturer's instructions. Cells were harvested at 24, 48, or 72 h after transfection to analyze the efficiency of overexpression.

RNA interference

Duplexes of siRNA were synthesized by Sangon Biotech (Shanghai, China). Specific siRNA sequences were listed in **Additional file 1: Table S6**. HepG2 cells with a density of 3×10^5 cells/well were passaged in a 6-well plate with Opti-MEM medium and transfected with siRNA using lipofectamine RNAiMAX (13778150, Invitrogen, USA) as described by the manufacturer. Cells were harvested 24, 48, or 72 h after transfection to analyze knockdown efficiency.

Quantitative real-time PCR (qPCR)

Total RNA was extracted from the cells or liver tissue using Trizol Reagent (15596018, Invitrogen, USA). 5 μg of total RNA was used for reverse transcription by a commercial kit (11123ES, Yeasen Biotech, China). qPCR was performed using the Top Green qPCR SuperMix (AQ132-24, TransGen, China) and analyzed on a QuantStudio 3 apparatus (Thermo Fisher Scientific). qPCR primer sequences were exhibited in **Additional file 1: Table S7**.

Lentivirus production

All liver transcription factors were cloned into Fu-tet-O lentiviral vectors (Addgene, #19778, USA). The Fu-Tet-O lentiviral vectors were provided by Dr. Zhao (College of Future Technology, Peking University). Briefly, coding DNA sequence of green fluorescent protein (GFP) and *Kif13b* or the polycistronic cassette from pc3.1 vectors were amplified by PCR and cloned into the Fu-tet-O vector using Gibson assembly according to the manufacturer's instructions (TransGene). Lentiviral vector

(15 μg) was co-transfected with pMDLg/pRRE, RSV/Rev, and VSV-G (5 μg each) into 293T cells (provided by Dr. Zhao, College of Future Technology, Peking University) using 75 μl PEI Max (1 mg/ml, PolySciences, USA) and 800 μl OptiMEM (Gibco, USA) in 10-cm dishes according to the manufacturer's instructions. After 12 h, the medium was changed, and viral supernatant was collected after an additional 36 h and filtered through 0.45 μm filters (Millipore, USA). The viruses were then stored at –80 °C for future use. For lentiviral gene transfer, the lentiviral-containing supernatants were collected, filtered through 0.45 μm filters, and concentrated 500-fold via ultra-centrifugation (120,000 × *g* for 2 h at 4 °C), and re-suspended in PBS with polybrene (8 μg/ml).

Measurement of cellular oxygen consumption rate (OCR)

The OCR was measured using the XF96 Analyzer (Seahorse Bioscience, USA). All procedures were carried out following the manufacture's guidelines. In summary, HepG2 cells transfected with scrRNA or siKif13b were seeded at a density of 6×10^3 cells/well on Seahorse XF96 cell culture microplate and incubated in low buffered non-bicarbonate assay medium (XF base medium with 2 mmol/L glutamine, 1 mmol/L sodium pyruvate, and 25 mmol/L glucose) for 1 h at 37 °C in a non-CO_2 incubator before analysis in an XFe 96 extracellular flux analyzer (Seahorse Bioscience, USA). The rate of oxygen consumption was measured for 3 periods with a mixing of 3 min in each cycle. Various inhibitors and activators were used at specific concentrations: oligomycin (2 μmol/L), carbonyl cyanide 4-(trifluoromethoxy)phenylhydrazone (FCCP, 1 μmol/L), antimycin A and rotenone (0.5 μmol/L). By utilizing these agents, the mitochondrial OCR was determined in different states including basal respiration, ATP production, maximal respiration, and spare respiration. The results were normalized to the corresponding total protein content per well.

Liquid chromatography-mass spectrometry (LC-MS) approach

Trypsin digestion

The stained protein bands were cut into pieces and washed with mass spectrometry water. The gels were detained with acetonitrile (ACN) until the gel turned white and then washed twice with mass spectrometry water for 10 min/wash. ACN was added to induce dehydration, which occurred until the colloidal particles turned white. Proteins on the gels were treated with 10 mmol/L Dithiothreitol (DTT) for 1 h at 37 °C and subsequently alkylated with 55 mmol/L iodoacetamide for 30 min in the dark. Add ACN again to dehydrate until the colloidal particles turn white, vacuum dry, and then add deionized water for cleaning; Add 50 mmol/L ammonium bicarbonate and incubate for 10 min, then add trypsin to fully contact the enzyme solution with the micelles. After the enzyme solution is

completely absorbed by the micelles, incubate at 37 °C overnight; the next day, centrifuge to collect the enzymatic hydrolysis supernatant.

LC-MS/MS analysis

Nanoflow LC-MS/MS analysis of tryptic peptides was conducted on a quadrupole Orbitrap mass spectrometer (Orbitrap Exploris™ 480, Thermo Fisher Scientific, Bremen, Germany) coupled to an EASY nLC 1200 ultra-high-pressure system (Thermo Fisher Scientific, Bremen, Germany) via a nano-electrospray ion source. The mass spectrometer was operated in "top-40" data-dependent mode, collecting mass spectrometry spectra in the Orbitrap mass analyzer (120,000 resolutions, 350 – 1500 m/z range) with an automatic gain control (AGC) target of 3E6 and a maximum ion injection time of 80 ms. The most intense ions from the full scan were isolated with an isolation width of 1.6 m/z. Following higher-energy collisional dissociation with a normalized collision energy (NCE) of 27, MS/MS spectra were collected in the Orbitrap (15,000 resolution) with an AGC target of 5E4 and a maximum ion injection time of 45 ms. To ascertain the identity of the proteins co-immunoprecipitated with KIF13B-GFP, a mass spectrometry analysis was conducted, employing GFP protein as a control.

RNA-seq data analysis

The human patients' bulk RNA-seq raw data GSE126848 were mapped to the human genome version hg38 using Hisat2 (version 2.2.1) [3] and other mouse bulk RNA-seq data were mapped to mouse genome version mm10. Then we submitted the mapped reads to the count function in HTSeq (version 2.0.5; https://htseq.readthedocs.io/en/latest/) [4] to calculate RNA-seq raw counts in each sample. The R package EdgeR (v3.38.4) [5] and DEseq2 (v1.36.0) [6] were used for differential expression analysis and the cut-off of 0.264 $\log_2$ fold change (FC) and 0.05 *P*-value were applied.

Pathway enrichment of differentially expressed genes

We submitted the differentially expressed gene sets to KOBAS [7] (http://bioinfo.org/kobas/) and used the Gene-list Enrichment function to perform pathway enrichment analysis in Gene Ontology (GO), Kyoto Encyclopedia of Genes and Genomes (KEGG), and REACTOME datasets, respectively. Pathways with a *P*-value < 0.05 were chosen as significantly enriched pathways.

Gene set enrichment analysis (GSEA) of differentially expressed genes

We first use the R function lfcShrink from DEseq2 to get shrunk $\log_2$ FC and sorted differentially expressed genes by shrunkLog_2 FC value. Then the R package ClusterProfiler (v4.4.4) [8] was used

to perform GSEA analysis based on MSigDB Mouse Collections GMT files. The cut-off of 0.05 *P*-value and 1.0 normalized enrichment score (NES) were applied to choose the significantly enriched pathway.

Genes correlation analysis

We performed Pearson correlation analysis in 57 samples in GSE126848 and got the Pearson correlation coefficient between *Kif13b* and other genes. The genes correlated with *Kif13b* were sorted by their Pearson correlation coefficient absolute value and we chose top 30 genes in each pathway-associated gene set. The cytoscape [9] (v3.10.2; https://cytoscape.org/) was used to visualize the genes co-expression network with origin node *Kif13b* and target node *Kif13b*-associated genes. The color of nodes was mapped to the Pearson correlation coefficient absolute value.

Single-cell/nucleus RNA-seq analysis

We collected the 10× profile of GSE212837 and GSE225381 from the Gene Expression Omnibus (GEO) dataset and used the Python package scanpy (v1.9.5) [10] was used for the downstream analysis. We filtered genes shown in lower than 10 cells and cells have lower than 1000 genes. Then we used scVI (v1.1.2) [11] to eliminate the batch effect. We annotated the clusters based on marker genes in the CellMarker dataset. The Python package pydeseq2 (v0.4.8) was used to normalize gene counts and perform different gene expression analyses in Hepatocytes. Genes with *P*-value < 0.05 were considered as different expression genes. We use the Deseq2 test method in GSE212837 data and the Mann-Whitney *U* test in GSE225381.

References

1. Lam SM, Li J, Sun H, Mao W, Lu Z, Zhao Q, et al. Quantitative lipidomics and spatial ms-imaging uncovered neurological and systemic lipid metabolic pathways underlying troglomorphic adaptations in cave-dwelling fish. Mol Biol Evol. 2022;39(4):msac050.
2. Charni-Natan M, Goldstein I. Protocol for primary mouse hepatocyte isolation. STAR Protoc. 2020;1(2):100086.
3. Zhang Y, Park C, Bennett C, Thornton M, Kim D. Rapid and accurate alignment of nucleotide conversion sequencing reads with HISAT-3N. Genome Res. 2021;31(7):1290-5.
4. Putri GH, Anders S, Pyl PT, Pimanda JE, Zanini F. Analysing high-throughput sequencing data in Python with HTSeq 2.0. Bioinformatics. 2022;38(10):2943-5.
5. Robinson MD, Mccarthy DJ, Smyth GK. edgeR: a Bioconductor package for differential expression analysis of digital gene expression data. Bioinformatics. 2010;26(1):139-40.

6. Love MI, Huber W, Anders S. Moderated estimation of fold change and dispersion for RNA-seq data with DESeq2. Genome Biol. 2014;15(12):550.
7. Bu D, Luo H, Huo P, Wang Z, Zhang S, He Z, et al. KOBAS-i: intelligent prioritization and exploratory visualization of biological functions for gene enrichment analysis. Nucleic Acids Res. 2021;49(W1):W317-25.
8. Yu G, Wang LG, Han Y, He QY. clusterProfiler: an R package for comparing biological themes among gene clusters. OMICS. 2012;16(5):284-7.
9. Shannon P, Markiel A, Ozier O, Baliga NS, Wang JT, Ramage D, et al. Cytoscape: a software environment for integrated models of biomolecular interaction networks. Genome Res. 2003;13(11):2498-504.
10. Wolf FA, Angerer P, Theis FJ. SCANPY: large-scale single-cell gene expression data analysis. Genome Biol. 2018;19(1):15.
11. Gayoso A, Lopez R, Xing G, Boyeau P, Valiollah Pour Amiri V, Hong J, et al. A Python library for probabilistic analysis of single-cell omics data. Nat Biotechnol. 2022;40(2):163-6.

Table S1 Sequences of sgRNAs

**sgRNAs**		**Sequences**
sgRNA1	M-Kif13b-E6A-gRNA up	TAGGAGGCTCAGGCCATCCTGCC
	M-Kif13b-E6A-gRNA down	AAACGGCAGGATGGCCTGAGCCT
sgRNA2	M-Kif13b-E6A-gRNA up	TAGGTGGTCATTTAGGATGAGTG
	M-Kif13b-E6A-gRNA down	AAACCACTCATCCTAAATGACCA

Kif13b kinesin family member 13b

Table S2 shRNA sequences

**Gene name**	**Species**	**Gene ID**	**Sequences**
Kif13b	Golden Syrian hamster	101833163	5'-GGCCAUUGAAGUAUAUGGACAUAAA-3'
AMPKα1	Mouse	105787	5'-GCAATCAAGCAGTTGGATTAT-3'

Kif13b kinesin family member 13b, *AMPKα1* AMP-activated catalytic subunit alpha 1

Table S3 Primer sequences for genotyping

Item	Sequences
Kif13b	Forward: ATTGTTCCCTGCACTATTACAA
	Reverse: GGGAAGTGTAGGTCTGAGCTAG
Kif13b flox up	Forward: GGTAAGGACTTTAGGCATTTATAC
	Reverse: CTGCAGGGTAAACAAAGTGA
Kif13b flox down	Forward: ATTGTTCCCTGCACTATTACAA
	Reverse: GGGAAGTGTAGGTCTGAGCTAG

Kif13b kinesin family member 13b

Table S4 MAFLD activity score

Histologic feature	Category	Score
Steatosis (%)	< 5	0
	5 – 33	1
	34 – 66	2
	> 66	3
Lobular inflammation	None	0
	< 2 foci per 200× field	1
	2 – 4 foci per 200× field	2
	> 4 foci per 200× field	3
Hepatocyte ballooning degeneration	None	
	Few balloon cells	
	Many balloon cells or prominent ballooning	
Sum of steatosis, lobular inflammation, and hepatocyte ballooning		MAFLD activity score (0 – 8)
Fibrosis grade	None	0
	Perisinusoidal or periportal	1
	Mild, zone 3, perisinusoidal	1A
	Moderate, zone 3, perisinusoidal	1B
	Portal/periportal	1C
	Perisinusoidal and portal/periportal	2
	Bridging fibrosis	3
	Cirrhosis	4

MAFLD metabolic dysfunction-associated fatty liver disease

Table S5 Primary antibodies

Antibody name	Product number	Source/purification	Producers	Nation
AMPKα	#2532	Rabbit polyclonal	Cell Signaling Technology	USA
Phospho-AMPKα Thr172	#2535	Rabbit monoclonal antibody	Cell Signaling Technology	USA
AMPKα1	ab271188	Rabbit monoclonal	Abcam	USA
Phospho-AMPKα1 Ser485	#2537	Rabbit monoclonal	Cell Signaling Technology	USA
Acetyl-CoA carboxylase	#3676	Rabbit monoclonal	Cell Signaling Technology	USA
Phospho-acetyl-CoA carboxylase (Ser79)	#11818	Rabbit monoclonal	Cell Signaling Technology	USA
Srebp1	ab28481	Rabbit polyclonal	Abcam	USA
Srebp2	ab30682	Rabbit polyclonal	Abcam	USA
GAPDH	10494	Rabbit polyclonal	Proteintech	USA
MT-CO1	BA2149	Rabbit polyclonal	Boster	China
UQCRC2	14742	Rabbit polyclonal	Proteintech	USA
NDUFS3	15066	Rabbit polyclonal	Proteintech	USA
SDHA	14865	Rabbit polyclonal	Proteintech	USA
Kif13b	SAB2101257	Rabbit polyclonal	Sigma	Germany

AMPKα AMP-activated catalytic subunit alpha, *Srebp1* sterol regulatory element binding protein 1, *Srebp2* sterol regulatory element binding protein 2, *GAPDH* glyceraldehyde-3-phosphate dehydrogenase, *MT-CO1* cytochrome c oxidase subunit 1, *UQCRC2* ubiquinol-cytochrome c reductase core protein 2, *NDUFS3* NADH-ubiquinone iron-sulfur protein 3, *SDHA* recombinant succinate dehydrogenase complex subunit A, *Kif13b* kinesin family member 13b

Table S6 Human *KIF13B* gene siRNA sequences

**Gene name**	**Sequences sense (5' – 3')**	**Anti-sense (5' – 3')**
siKIF13B-1	CCUCCAUGAAGAACGAGAAUATT	UAUUCUCGUUCUUCAUGGAGGTT
siKIF13B-2	CCCAGUAAUACGAUCAUACUUTT	AAGUAUGAUCGUAUUACUGGGTT
siKIF13B-3	GCCUUGAAGAUCUGCGACAAATT	UUUGUCGCAGAUCUUCAAGGCTT

KIF13B kinesin family member 13B

Table S7 Mouse qPCR primer sequences

Gene name	Forward primer	Reverse primer
Kif13b	AACGAACCCAGAAAGAGGA	GCTTGTGACAGCCAGTTTA
Srebp1a	GGCCGAGATGTGCGAACT	TTGTTGATGAGCTGGAGCATGT
Srebp1c	GGAGCCATGGATTGCACATT	GGCCCGGGAAGTCACTGT
Srebp2	GCGTTCTGGAGACCATGGA	ACAAAGTTGCTCTGAAAACAAATCA
Scap	ATTTGCTCACCGTGGAGATGTT	GAAGTCATCCAGGCCACTACTAATG
Insig1	TCACAGTGACTGAGCTTCAGCA	TCATCTTCATCACACCCAGGAC
Insig2	CGGAAGATGCTGGAACCTGACT	GCACACGCACACCACAGTAGT
Lxrα	TCTGGAGACGTCACGGAGGTA	CCCGGTTGTAACTGAAGTCCTT
Abca1	AGGCTGCTGCTGTGGAAGAATC	CAGGCGAGACACGATGGACTTG
Abcg5	TGGATCCAACACCTCTATGCTAAA	GGCAGGTTTTCTCGATGAACTG
Abcg8	TGCCCACCTTCCACATGTC	ATGAAGCCGGCAGTAAGGTAGA
Apoa1	AGACAGCGGCAGAGACTATGTG	TCCCAGAAGTCCCGAGTCAATG
Hmgcr	TGAGCAGCGACATCATCATCCT	GGCATTCCACAAGAGCGTCAAG
Hmgcs	ACACCTGCTCACCTGCTCTCA	TTGTGCGTTCCATCAGCCTCTG
Ldlr	AGGCTGTGGGCTCCATAGG	TGCGGTCCAGGGTCATCT
Pcsk9	CAGGCGGCCAGTGTCTATG	GCTCCTTGATTTTGCATTCCA
Cyp7a	GCTGTGCTCTGAAGTTCGGATC	TTCTGTGTCCAAATGCCTTCGC
Acly	CTGCTTGTGGGTGTGGACGAAA	GCCGCCAAGTCAAGGATGTAGA
Acs	TGATTGACATTCGGCAGTA	CTGACATCGTCGTAGTAGTA
Acc1	GCAGACCACTATGTTCCA	GCCTTCAGACCATCATCC
Scd1	GGTCATCCCATCGCCTGCTCTA	TGGTGGTGGTGGTCGTGTAAGA
Fans	CTCCTGAAGCCGAACACCTCTG	ACCTTGCTCCTTGCTGCCATC
Gapt1	GGACTGGGTTGACTGTGGGTTC	GCCATCCTCTGTGCCTTGTGT
Tnfα	GCCACCACGCTCTTCTGTCTAC	GACGGCAGAGAGGAGGTTGACT
Il6	GACCTGTCTATACCACTTCAC	GTGCATCATCGTTGTTCATAC
Il1β	TGGACCTTCCAGGATGAGGACA	GTTCATCTCGGAGCCTGTAGTG
Il10	TGCTGCCTGCTCTTACTGACTG	CTGCTCCACTGCCTTGCTCTT
Mpo	AGTTGTGCTGAGCTGTATGGA	CGGCTGCTTGAAGTAAAACAGG
Ly6c	GCAGTGCTACGAGTGCTATGG	ACTGACGGGTCTTTAGTTTCCTT
Mcp1	CCACTCACCTGCTGCTACTCAT	CACTGTCACACTGGTCACTCCT
Tgfβ	GGACCGCAACAACGCCATCTAT	TTCAGCCACTGCCGTACAACTC

αsma	TGGTGTGCGACAATGGCTCTG	CAGTTGGTGATGATGCCGTGTT
Col1α	TCCTTCTGGTCCTCGTGGTCTC	AGCCTCGGTGTCCCTTCATTCC
Mttp	CTCTTGGCAGTGCTTTTTCTCT	GAGCTTGTATAGCCGCTCATT
Apob	AAGCACCTCCGAAAGTACGTG	CTCCAGCTCTACCTTACAGTTGA
Fabp1	GGAAGGACATCAAGGGGGTG	TCACCTTCCAGCTTGACGAC
Pparα	AGAGCCCCATCTGTCCTCTC	ACTGGTAGTCTGCAAAACCAAA
Cpt1	CTCCGCCTGAGCCATGAAG	CACCAGTGATGATGCCATTCT
Cpt2	CAGCACAGCATCGTACCCA	TCCCAATGCCGTTCTCAAAAT
Acat1	CAGGAAGTAAGATGCCTGGAAC	TTCACCCCCTTGGATGACATT
Ehhadh	ACAGCGATACCAGAAGCCAGTG	AGAGCAACAGGAACTCCAACGA
Acadm	AGGGTTTAGTTTTGAGTTGACGG	CCCCGCTTTTGTCATATTCCG
β-actin	TGTGCTGTCCCTGTATGCCTCT	GGAACCGCTCGTTGCCAATAGT

Kif13b kinesin family member 13b, *Srebp1a* sterol regulatory element binding protein 1a, *Srebp1c* sterol regulatory element binding protein 1c, *Srebp2* sterol regulatory element binding protein 2, *Scap* sterol regulatory element binding protein cleavage-activating protein, *Insig1* insulin induced gene 1, *Insig2* insulin induced gene 2, *Lxrα* liver X receptor alpha, *Abca1* ATP-binding cassette transporter A1, *Abcg5* ATP binding cassette transporter G5, *Abcg8* ATP binding cassette transporter G8, *Apoa1* apolipoprotein A1, *Hmgcr* 3-hydroxy-3-methylglutaryl-CoA reductase, *Hmgcs* 3-hydroxy-3-methylglutaryl coenzyme A (HMG-CoA) synthase, *Ldlr* low density lipoprotein receptor, *Pcsk9* proprotein convertase subtilisin/kexin type 9, *Cyp7a* cholesterol 7α-hydroxylase, *Acly* ATP citrate lyase, *Acs* acyl-CoA synthetase, *Acc1* acetyl-CoA carboxylase 1, *Scd1* stearoyl-CoA desaturase 1, *Fans* fatty acid synthase, *Gapt1* glycerol transporter 1, *Tnfα* tumor necrosis factor alpha, *Il6* interleukin-6, *Il1β* interleukin-1 beta, *Il10* interleukin-10, *Mpo* myeloperoxidase, *Ly6c* lymphocyte antigen 6 complex, locus C1, *Mcp1* monocyte chemotactic protein-1, *Tgfβ* transforming growth factor-β, *αsma* alpha-smooth muscle actin, *Col1α* collagen type I alpha 1, *Mttp* microsomal triglyceride transfer protein, *Apob* apolipoprotein B, *Fabp1* fatty acid binding protein 1, *Pparα* peroxisome proliferator activated receptor alpha, *Cpt1* carnitine palmitoyltransferase 1, *Cpt2* carnitine palmitoyltransferase 2, *Acat1* acetyl-CoA acetyltransferase 1, *Ehhadh* 3-hydroxyacyl-CoA dehydrogenase, *Acadm* acyl-CoA dehydrogenase medium chain, *β-actin* beta-actin

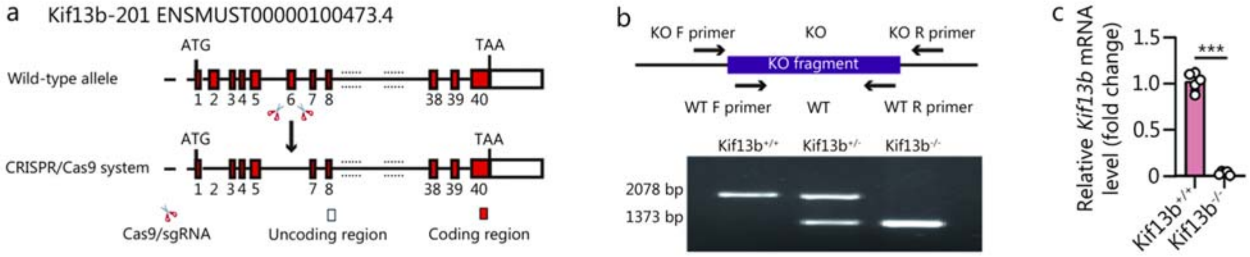

Fig. S1 Construction of a *Kif13b* knockout mouse model. **a** A schematic representation of a *Kif13b* whole-body knockout (Kif13b$^{-/-}$) mouse has been constructed. **b** Genotyping of F2 generation from wild-type (WT, Kif13b$^{+/+}$), *Kif13b* heterozygous (Kif13b$^{+/-}$), and homozygous knockout (Kif13b$^{-/-}$) mice. **c** *Kif13b* mRNA level in mouse livers (n = 5). Data are means ± SEM. ***P < 0.001. P-values were calculated using an unpaired two-tailed Student's t-test. KO knockout, Kif13b kinesin family member 13b

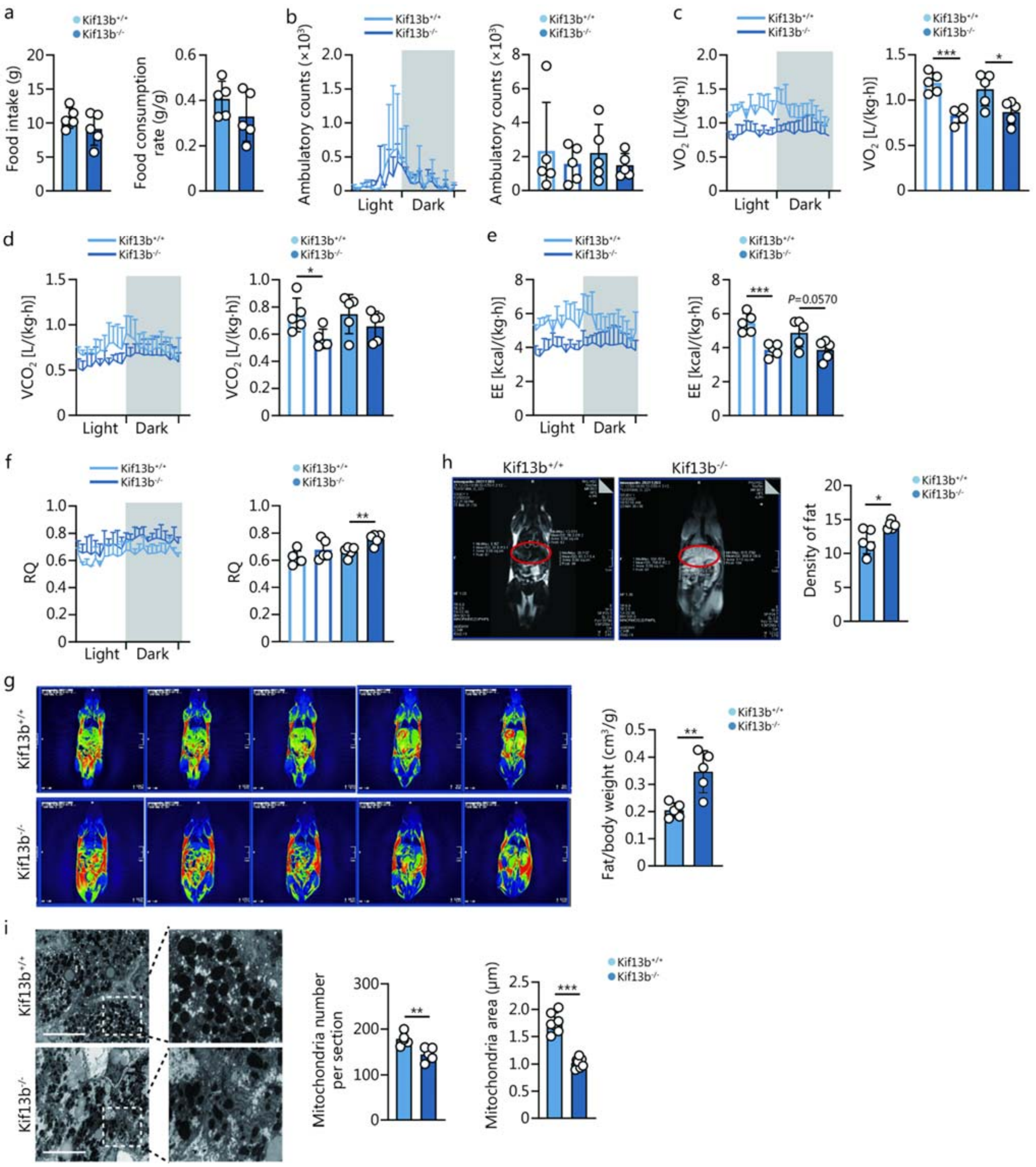

Fig. S2 *Kif13b* deficiency disrupts energy metabolism in mice on a Western diet (WD). Eight-week-old male mice were fed with a WD for 16 weeks and then placed in metabolic cages for the evaluation of various metabolic parameters. **a** Food intake and food consumption rate. **b** Monitoring and quantification of ambulatory counts during the light and dark periods. **c** Monitoring and quantification of oxygen consumption (VO_2) during both light and dark periods. **d** Measurement and quantification of carbon dioxide production (VCO_2) volume during the light and dark periods. **e** Measurement and quantification of energy expenditure (EE) during the light and dark periods. **f**

Recording and quantification of respiratory quotient (RQ) during the light and dark periods. **g** Visualization and quantification of body fat ratio using magnetic resonance imaging (MRI). **h** Visualization and quantification of fat density using MRI. **i** Observation and quantification of the number and area of mitochondria in liver tissues using an electron microscope. Scale bars = 10 μm. *n* = 5. Data are means ± SEM. **P*< 0.05, ***P* < 0.01, ****P* < 0.001. *P*-values were calculated using an unpaired two-tailed Student's *t*-test. Kif13b kinesin family member 13b

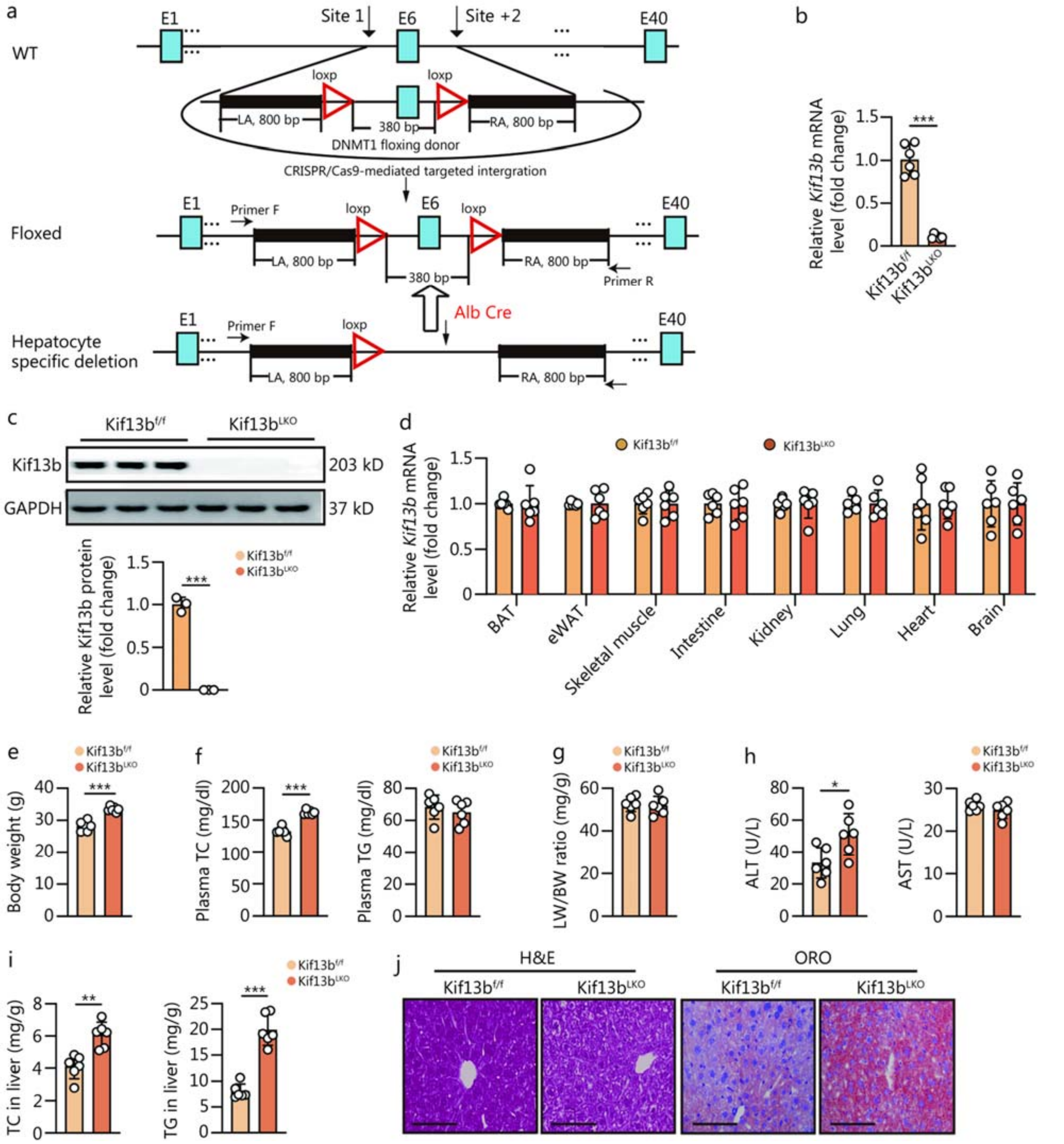

Fig. S3 Hepatic *Kif13b* deletion elicits hepatic steatosis. **a** A schematic representation of a liver-specific *Kif13b* knockout (Kif13b^{LKO}) mouse has been constructed. **b** *Kif13b* mRNA level in mouse livers. **c** Kif13b protein level in mouse livers. **d** The *Kif13b* mRNA expression in brown adipose tissue (BAT), epididymis white adipose tissue (eWAT), skeletal muscle, intestine, kidney, lung, heart, and brain of 6-month-old male Kif13b$^{f/f}$ and Kif13b^{LKO} mice. Eight-week-old male Kif13b$^{f/f}$ and Kif13b^{LKO} mice were fed with a chow diet (CD) for 16 weeks. **e** Body weight of indicated mice. **f** Plasma total cholesterol (TC) and triglyceride (TG). **g** The ratio of liver weight to body weight (LW/BW). **h** Plasma alanine aminotransferase (ALT) and aspartate aminotransferase

(AST) levels. **i** Liver TC and TG contents. **j** Representative histological images of liver cryo-sections with H&E staining and oil red O (ORO) staining from $Kif13b^{f/f}$ and $Kif13b^{LKO}$ mice. Scale bars = 100 μm. $n = 6$. Data are means ± SEM. $^{*}P < 0.05$, $^{**}P < 0.01$, $^{***}P < 0.001$. P-values were calculated by unpaired two-tailed Student's t-test. Kif13b kinesin family member 13b

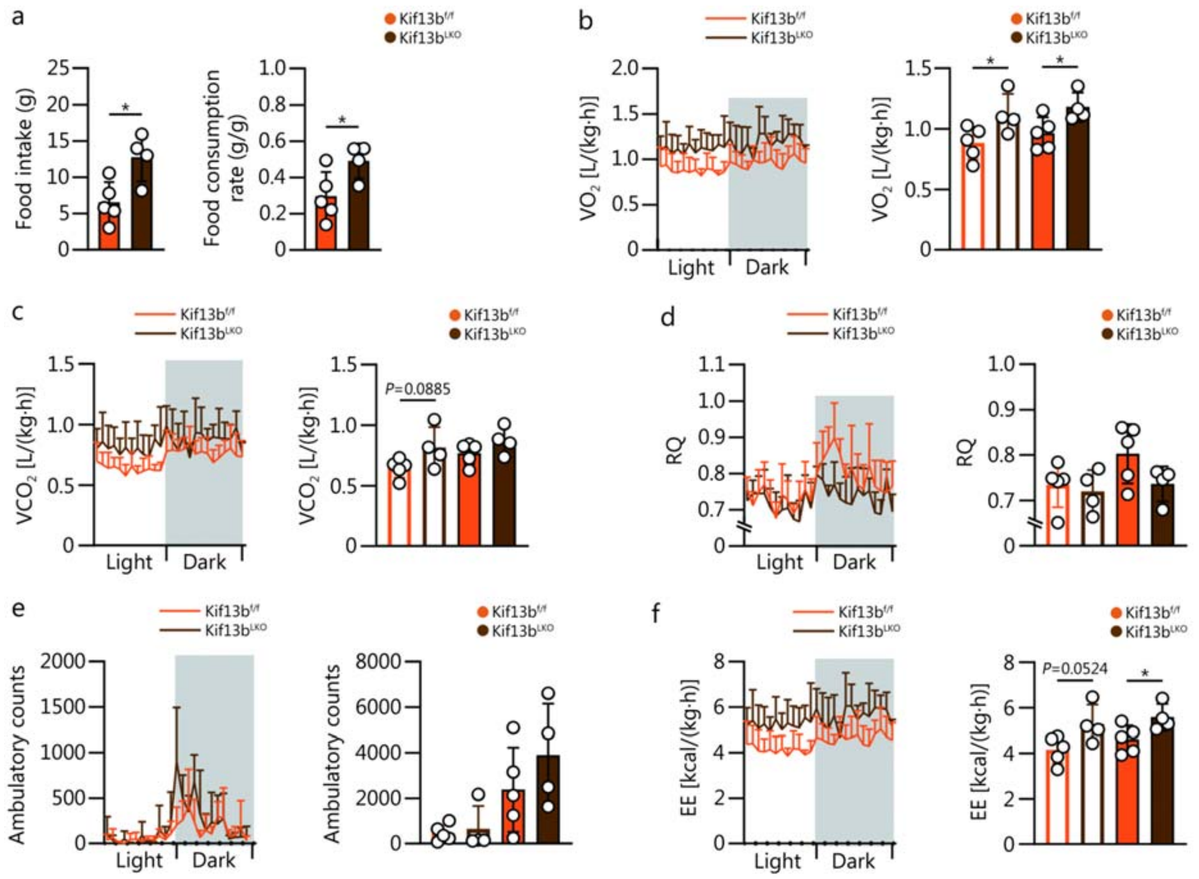

Fig. S4 Energy metabolism in liver-specific *Kif13b*-deficient mice. Six-month-old male Kif13b$^{f/f}$ (n = 5) and Kif13b^{LKO} (n = 4) mice were accommodated in metabolic cages for the assessment of various metabolic parameters. **a** Assessment of mice food intake and food consumption rate. **b** Monitoring and quantification of oxygen consumption (VO_2) during both light and dark periods. **c** Measurement of carbon dioxide production (VCO_2) volume during light and dark periods. **d** Recording and quantification of respiratory quotient (RQ) during both light and dark periods. **e** Monitoring and quantification of ambulatory counts during both light and dark periods. **f** Measurement and quantification of energy expenditure (EE) during both light and dark periods. n = 4-5. Data are means ± SEM. *$P < 0.05$. P-values were calculated using an unpaired two-tailed Student's t-test. Kif13b kinesin family member 13b

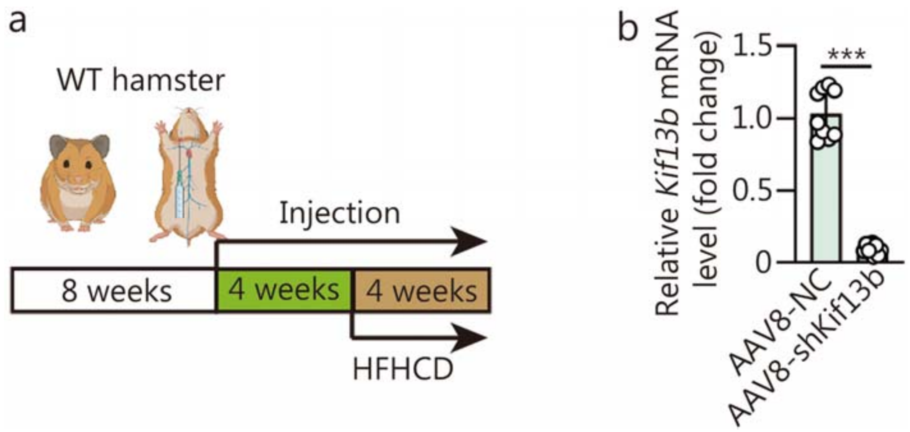

Fig. S5 A hepatic Kif13b knockdown hamster model was constructed using adeno-associated virus vector (AAV) 8. **a** Eight-week-old WT hamsters received jugular vein injections of AAV8-shKif13b to deplete *Kif13b* from their livers, with AAV8-negative control (NC) as a control. All hamsters were then placed on a CD for 4 weeks, followed by a high-fat and high-cholesterol diet (HFHCD) for another 4 weeks. **b** *Kif13b* mRNA level in hamster livers. $n = 8$. Data are means ± SEM. $^*P < 0.05$. *P*-values were calculated using an unpaired two-tailed Student's *t*-test. Kif13b kinesin family member 13b

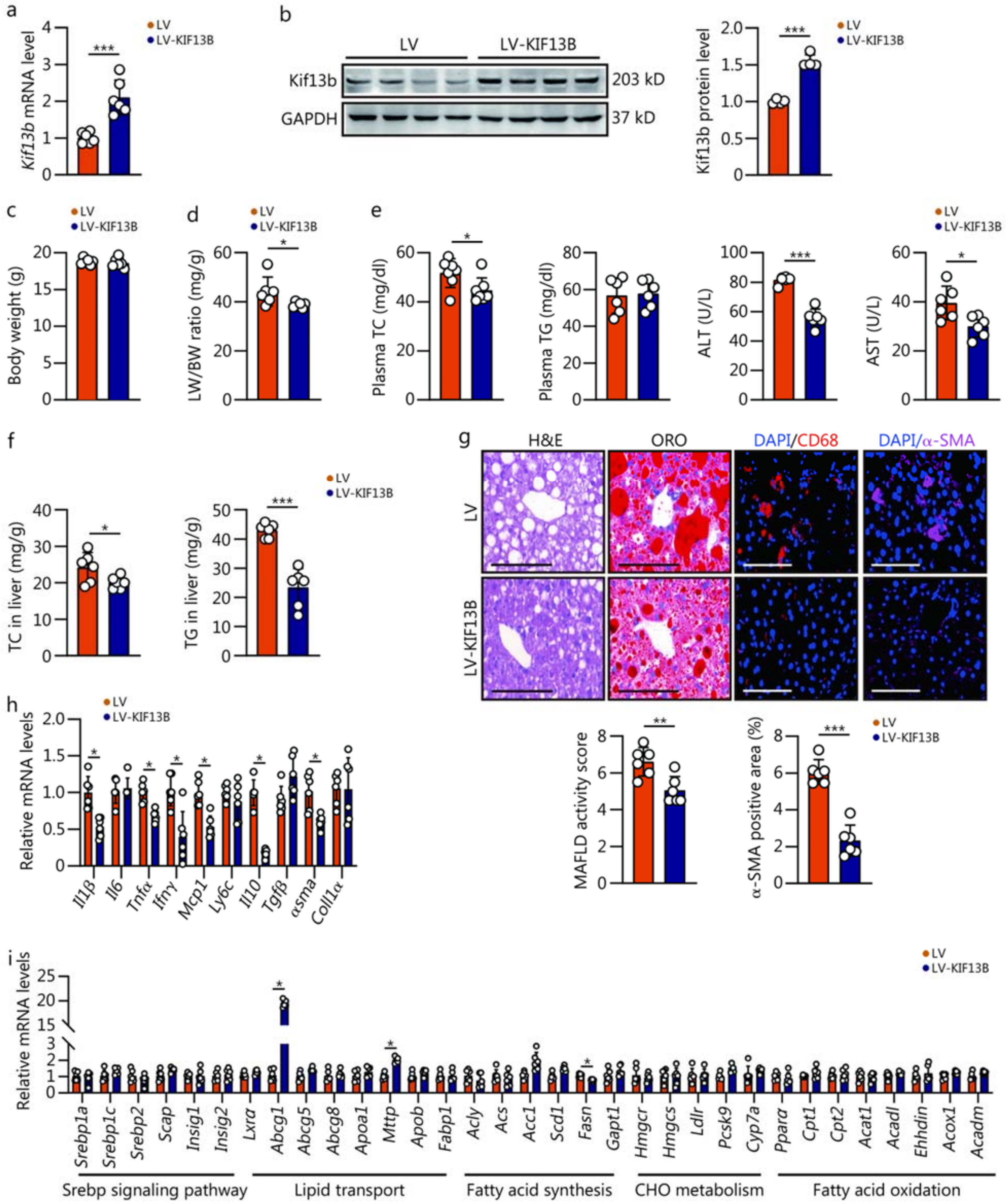

Fig. S6 Hepatic *KIF13B* overexpression prevents metabolic dysfunction-associated steatohepatitis (MASH) in methionine-choline-deficient diet (MCD)-fed mice. Eight-week-old male WT mice were subjected to a 3-week diet of MCD feeding. Meanwhile, they were administered lentivirus (LV) or LV-KIF13B, through the portal vein. **a** *Kif13b* mRNA level in mouse livers. **b** Representative Western blotting images and quantification of liver samples. **c** Body weight. **d** The ratio of liver weight to body weight (LW/BW). **e** Plasma total cholesterol (TC), triglyceride (TG), alanine

aminotransferase (ALT), and aspartate aminotransferase (AST) levels. **f** Liver TC and TG contents. **g** Representative histological images of liver cryo-sections with H&E staining, oil red O (ORO) staining, and immunofluorescence staining of CD68 and α smooth muscle actin (α-SMA). Scale bars = 100 μm. Histological scoring of MAFLD activity score and hepatic collagen area were calculated. **h** mRNA levels of proinflammatory cytokines and fibrosis markers of liver samples. **i** Analysis of the mRNA expression levels of genes associated with the Srebp signaling pathway, lipid transport, fatty acid synthesis, cholesterol (CHO) metabolism, and fatty acid oxidation of liver samples. $n = 6$. Data are means ± SEM. *$P < 0.05$, **$P < 0.01$, ***$P < 0.001$. P-values were calculated by unpaired two-tailed Student's t-test. Kif13b kinesin family member 13b, MAFLD metabolic dysfunction-associated fatty liver disease

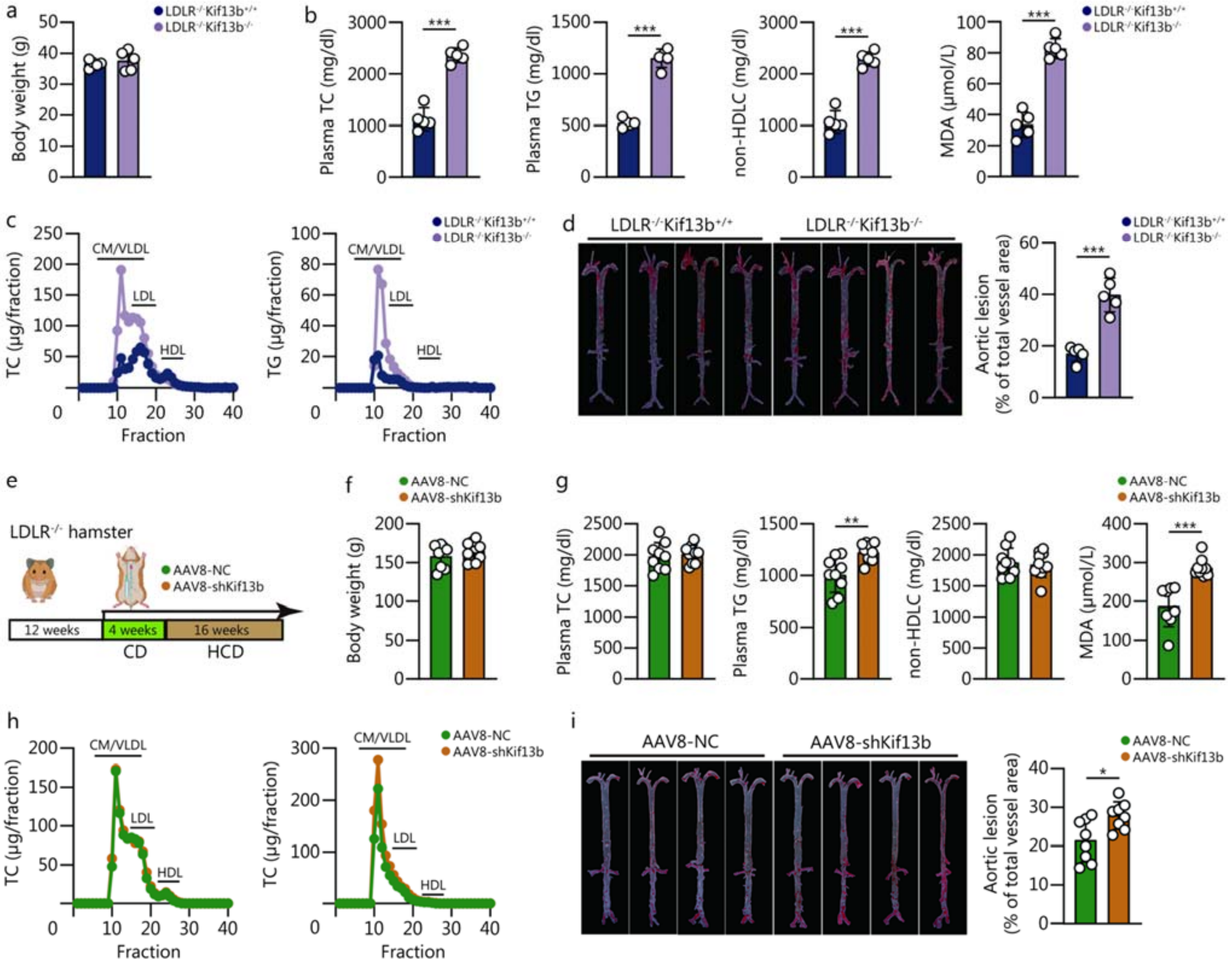

Fig. S7 Depletion of *Kif13b* predisposes LDLR$^{-/-}$ mice and hamsters to atherosclerosis. Sixteen-week-old male LDLR$^{-/-}$Kif13b$^{+/+}$ and LDLR$^{-/-}$Kif13b$^{-/-}$ mice were placed on a Western diet (WD) for 12 weeks. **a** Body weight. **b** Plasma levels of total cholesterol (TC), triglyceride (TG), non-high-density lipoprotein cholesterol (non-HDLC), and malondialdehyde (MDA) at the end of the experiment. **c** Analysis of TC and TG distribution conducted through fast protein liquid chromatography (FPLC) in mice. **d** Representative oil red O staining images depicting the en face of the aortas and quantification of aortic lesions across the entire aorta. *n* = 5. **e** Twelve-week-old LDLR$^{-/-}$ hamsters were subjected to jugular vein injection with either AAV8-negative control (NC) or AAV8-shKif13b. They were then maintained on a chow diet (CD) for 4 weeks, followed by a switch to a high-cholesterol diet (HCD) for an additional 16 weeks. **f** Body weight. **g** Plasma levels of TC, TG, non-HDLC, and MDA at the end of the experiment. **h** Analysis of TC and TG distribution conducted through FPLC in hamsters. **i** Representative images depicting the en face of the aorta and quantification of aortic lesions across the entire aorta. *n* = 8 – 9. Data are means ± SEM. *$P < 0.05$, **$P < 0.01$, ***$P < 0.001$. *P*-values were calculated by unpaired two-tailed Student's

t-test. Kif13b kinesin family member 13b, CM/VLDL chylomicron/very-low-density lipoprotein, HDL high-density lipoprotein

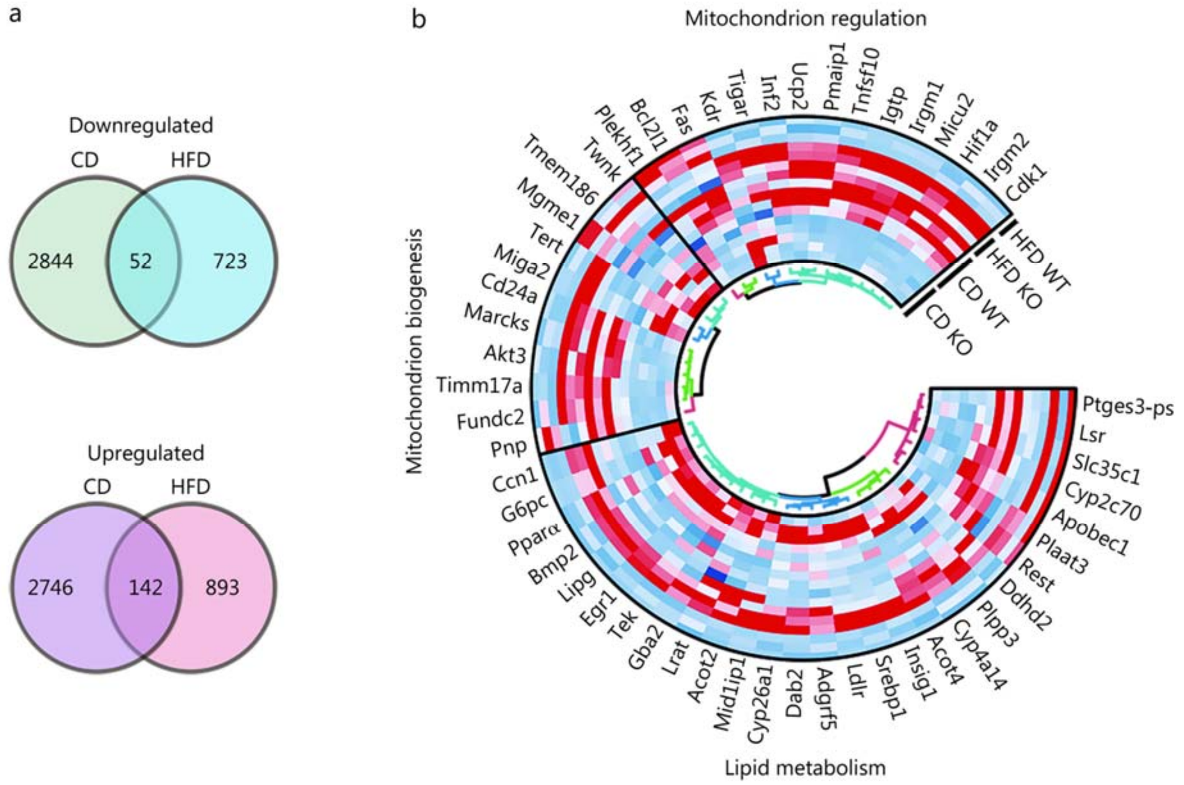

Fig. S8 Kif13b regulates lipid synthesis and mitochondrial function in the mouse liver. **a** The RNA sequencing Venn diagram depicts the differentially expressed genes (DEGs) in the livers of male Kif13b$^{+/+}$ and Kif13b$^{-/-}$ mice fed with chow diet (CD) (n = 5) and high-fat diet (HFD) (n = 3). The upper section of the diagram displays the downregulated genes, while the lower section depicts the upregulated genes in the Kif13b$^{-/-}$ mice liver. **b** Heatmap of DEGs in mitochondrion regulation, mitochondrion biogenesis, and lipid metabolism pathways [from Gene Ontology (GO) database] associated gene sets. Expression levels were normalized as Z-score

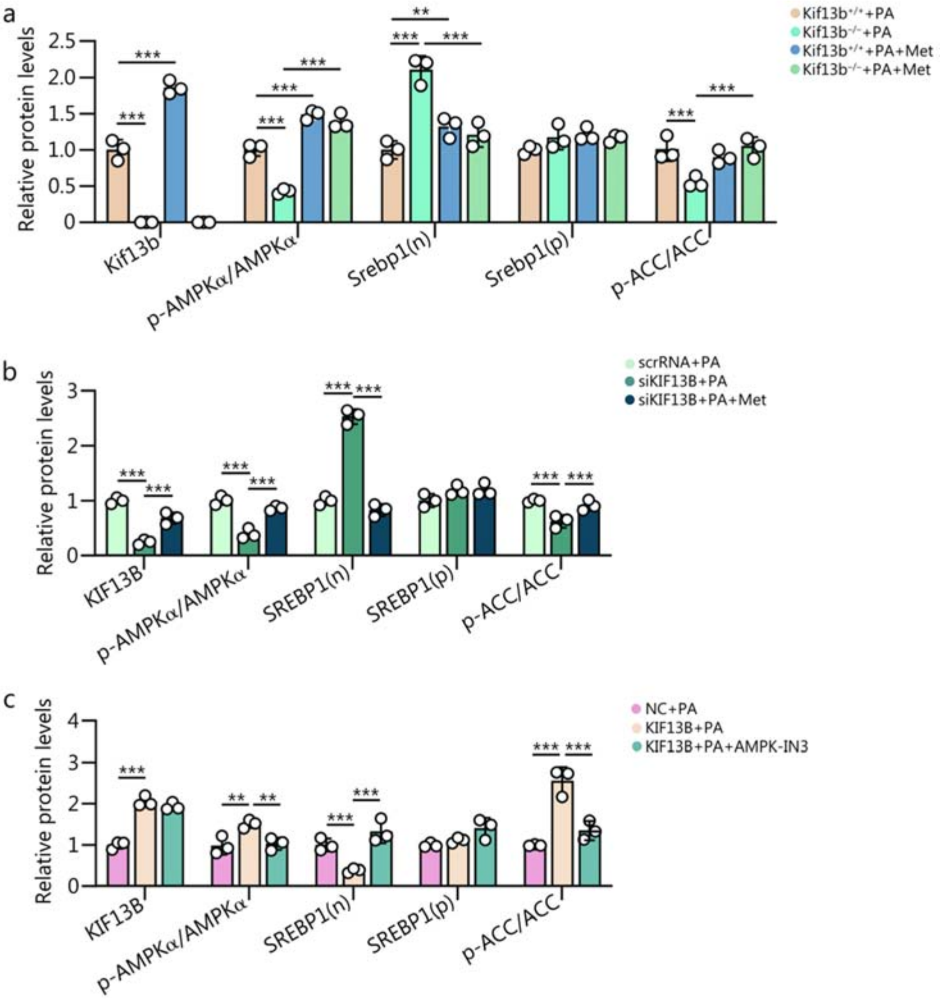

Fig. S9 Kif13b orchestrates lipid metabolism and mitochondrial function through AMPKα. Primary mouse hepatocytes from $Kif13b^{+/+}$ and $Kif13b^{-/-}$ mice were starved 12 h and then exposed to palmitic acid (PA) at a concentration of 300 μmol/L with or without metformin (Met) at 2 mmol/L for 24 h. **a** Relative quantification of Kif13b, p-AMPKα/AMPKα, Srebp1(n), Srebp1(p), and p-ACC/ACC. In HepG2 cells, transfection with scrambled RNA (scrRNA) or siKIF13B occurred for 48 h, followed by exposure to 300 μmol/L PA and 2 mmol/L Met for a further 24 h, after 12 h of starvation. **b** Relative quantification of Kif13b, p-AMPKα/AMPKα, Srebp1(n), Srebp1(p), and p-ACC/ACC. HepG2 cells were transfected with a negative control (NC) or KIF13B plasmid for 48 h and treated with 300 μmol/L PA and 110 nmol/L AMPK-IN3 (an AMPKα inhibitor) for a further 24 h, after 12 h of starvation. **c** Relative quantification of Kif13b, p-AMPKα/AMPKα, Srebp1(n), Srebp1(p), and p-ACC/ACC. Kif13b kinesin family member 13b, AMPKα AMP-activated catalytic subunit α, Srebp1 sterol regulatory element binding protein 1, ACC acetyl-CoA carboxylase

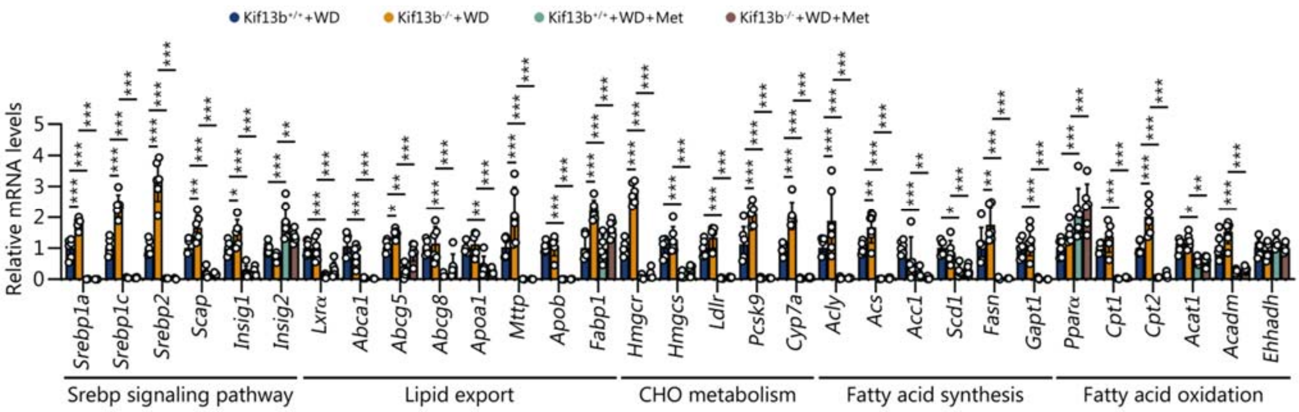

Fig. S10 Metformin improves *Kif13b* knockout-induced liver injury. Eight-week-old male mice were subjected to a 20-week Western diet (WD) and received vehicle or metformin (Met) every 2 d via gavage for 8 weeks. Analysis of the mRNA expression levels of genes associated with the Srebp signaling pathway, lipid export, cholesterol (CHO) metabolism, fatty acid synthesis and fatty acid oxidation of liver samples from mice. $n = 6$. Data are means ± SEM. *$P < 0.05$, **$P < 0.01$, ***$P < 0.001$. P-values were calculated using two-way ANOVA followed by Tukey's test. Kif13b kinesin family member 13b

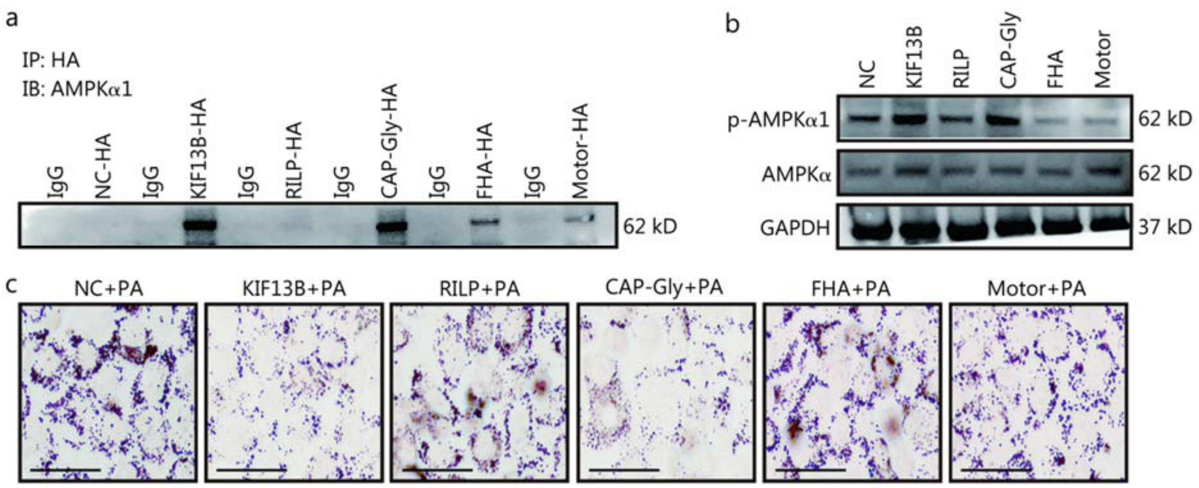

Fig. S11 Identification of CAP-Gly domain of Kif13b as the binding site for AMPKα1. HepG2 cells were transfected with negative control (NC)-HA, KIF13B-HA, RILP-HA, CAP-Gly-HA, FHA-HA, or Motor-HA plasmid for 48 h, followed by exposure to 300 μmol/L palmitic acid (PA) for another 24 h, after 12-hour starvation. **a** Immunoblotting of HA-AMPKα1 in co-immunoprecipitation (Co-IP) assays from transfected HepG2 cell lysates. **b** Immunoblotting of p-AMPKα1, AMPKα and GAPDH from transfected HepG2 cell lysates. **c** Representative images of oil red O (ORO) staining. Scale bars = 50 μm. HA Hemagglutinin Tag, RILP Rab interacting lysosomal protein-like 1 and 2 (Rilpl1 and Rilpl2) domain, CAP-Gly cytoskeleton-associated protein glycine-rich domain, FHA forkhead associated domain, Kif13b kinesin family member 13b
